# Pharmacological Effects of Humic Substances and Their Signaling Mechanisms

**DOI:** 10.3390/molecules31010114

**Published:** 2025-12-29

**Authors:** Maria V. Zykova, Evgenia S. Trofimova, Lyudmila A. Azarkina, Tatyana V. Lasukova, Dmitrii A. Mihalyov, Larisa A. Drygunova, Marina G. Danilets, Anastasia A. Ligacheva, Andrey V. Tsupko, Sergey R. Bashirov, Mikhail V. Belousov

**Affiliations:** 1 Faculty of Pharmacy, Siberian State Medical University, Tomsk 634050, Russia; trofimova_es@pharmso.ru (E.S.T.); ludmila_logvinova@mail.ru (L.A.A.); tlasukova@mail.ru (T.V.L.); diman021999@gmail.com (D.A.M.); l_drygunova@mail.ru (L.A.D.); frikins@my.com (A.V.T.); bars-tomsk@rambler.ru (S.R.B.); mvb63@mail.ru (M.V.B.); 2 Goldberg Research Institute of Pharmacology and Regenerative Medicine, Tomsk 634050, Russia; m.danilets@mail.ru (M.G.D.); vittelli@mail.ru (A.A.L.)

**Keywords:** humic substances, pharmacological effects, signaling mechanisms, immunotropic action, antioxidant protection, antiviral effect, detoxification, regeneration, adaptogenic, humic bionanomaterials

## Abstract

This comprehensive review presents the results of an in-depth analytical literature search on the biological activity of humic substances and their possible pharmacological mechanisms of action. The unique chemical structure of humic substances has determined their widespread use in many economic sectors, including medicine. Thanks to modern advances in pharmaceuticals, pharmacology, and toxicology, it has been possible to demonstrate the multifaceted biological activity of humic substances and, consequently, the possibility of using them to treat and prevent many infectious and non-infectious pathologies, including diseases considered incurable. The article presents data on their immunotropic, antibacterial, antiviral (including HIV), antitumor, antioxidant and antiradical, cardiotropic, hepatoprotective, regenerative, detoxifying, and adaptogenic effects; their influence on the intestinal microbiome; studies of the toxic properties of humic substances and the safety of their use in medicine; and the current trend of using humic substances as unique matrices for creating next-generation bionanomaterials. An analysis of data on the intracellular mechanisms that play a key role in the implementation of the effects of humic substances is conducted. Thus, the natural genesis of humic substances, their multifaceted biological activity, and the absence of toxic and allergenic properties explain the growing interest of scientists from all over the world in their study.

## 1. Introduction

Humic substances (HS) are currently of great interest for practical medical applications, and we are seeing increasing market growth in the supply of various HS-based food products, dietary supplements, and pharmaceuticals every year. The pharmacological effects of HS may depend on the type of raw material source (peat, coal, mumie) and its geographical origin, the technology used to produce humic products, and the chemical properties of the HS structure [1,2], and therefore, there is a vast diversity of scientific data on the biological properties of HS. This comprehensive review is devoted to systematizing the information on the experimentally established pharmacological effects of HS and their possible signaling mechanisms.

Humic substances are a group of ubiquitous dark-colored polyfunctional organic substances that are formed over a long period of time from components of biocenoses, mainly plants, as a result of their oxidative destruction occurring in a waterlogged environment with the direct participation of microbiota and/or atmospheric oxygen [3]. Humic substances are a necessary component of all metabolic processes in nature and the most thermodynamically stable form of preservation of organic compounds in the biosphere [3]. The synthesis of HS in many natural objects is a unique long-term biospheric process, in which from 0.6 to 2.5 × 10^9^ tons of carbon participate annually [4]. Moreover, the process of gradual transformation of plant organisms, called humification, involves flora inhabitants growing in any natural and climatic zone [3,4,5]. The result of such transformation of plant tissue components is the emergence of a unique class of organic compounds—HS, which ensure the existence of modern life forms and encode in their composition the conditions of the period of their formation. Humification regulates the balance between mineralization and conservation of organic remains.

Humic substances are cosmopolitan, they are found in various caustobioliths (peat, bottom sediments, coal, shale, soil, etc.) and are their basic components. In terms of structure and elemental content, HSs are stochastic and lack a defined chemical structure. They represent a unique, stabilized form of organic matter, uncontrolled by the conditions of the biological code and consistent only with the laws of thermodynamics [5].

Humic substances are classified [3] into humic acids (HA)—the dominant high-molecular fraction of HS, soluble in alkaline and insoluble in strongly acidic (pH = 1–2) solutions; fulvic acids (FA)—the HS fraction soluble in the entire pH range; hymatomelanic acids—the HS fraction that passes into solution during the treatment of fresh HA sediment with ethyl alcohol; humin—a substance insoluble in acids, alkalis and organic solvents.

Humic substances can be considered either biopolymer macromolecules or colloidal micelles or supramolecules composed of relatively small molecules. This ambiguity in the structure of HS is due to their varying chemical behavior depending on environmental conditions. Thus, HSs are complex molecular assemblies, including structures with polymeric and supramolecular characteristics and not having a strictly constant chemical composition, i.e., they are so-called “molecular assemblies” [6]. It is believed that the unique chemical feature of HS is their extreme structural heterogeneity, which contributes to their resistance to biodegradation [6,7].

The process of HS formation (humification) is one of the most complex and controversial issues. As a result of many years of research, several key scientific theories have been formed. Fundamental studies by F.J. Stevenson [3], M.H. Engel and S.A. Macko [4], G.R. Aiken [5] show two main classical theories. Firstly, this is the theory of biochemical oxidation, the so-called lignin-protein theory. According to this theory, HSs are formed as a result of various chemical reactions and microbial transformation of the structural components of plant tissues, primarily lignin and proteins [3,5]. Thus, easily degradable components (polysaccharides) are consumed by microorganisms, and more stable components (lignin) undergo oxidation, demethylation and cleavage with the formation of phenolic structures. Lignin transformation products then undergo condensation reactions with amino acids and peptides (protein degradation products), forming complex HS macromolecules. Secondly, there is the theory of polymelanoidins, the so-called sugar-amine condensation. This theory is based on the Maillard reaction between reducing sugars and amino acids [3,4] without the participation of lignin. This reaction results in the formation of melanoidins (dark-colored nitrogen-containing polymers) with properties similar to HS. This theory demonstrates the possibility of HS formation in environments poor in lignin but rich in microbial biomass.

There is also a modern view in the field of humification theory based on supramolecular association and stochastic synthesis [6,8,9,10]. Experimental data obtained using the method of ultra-high-resolution ion cyclotron mass spectrometry with Fourier transform (FTICR MS) show that HSs are complex mixtures of thousands of relatively small and diverse molecules that are held together by non-covalent interactions (hydrogen bonds, hydrophobic and electrostatic forces) into dynamic supramolecular assemblies [6,8,9,10]. The process of formation of such assemblies is described as stochastic synthesis or unprogrammed recombination of biomacromolecule degradation products [6]. A variety of precursors are involved in this process—not only lignins and proteins, but also lipids, tannins, carbohydrates and microbial metabolites. Their random reactions and abiotic condensation lead to the formation of a “molecular ensemble” in which the structures most resistant to further decomposition are preserved [6]. The main chemical mechanism of such molecular diversity is oxidative dearomatization. According to N. Hertkorn [8] and I.V. Perminova [6], this process transforms aromatic precursors (e.g., from lignin) into alicyclic and aliphatic structures with a large number of oxygen-containing functional groups, which is characteristic of HS.

It should be noted that the modern interpretation of humification processes does not reject classical theories, but allows for a more accurate interpretation of the predominance of a particular humification pathway depending on environmental conditions and the initial organic material [3,6]. For example, in the works of A.Y. Zherebker et al. [9,10], it was shown that HS from coal and peat have significant differences in their molecular composition, which depends on the different conditions of their structure formation. Thus, coal HSs are more aromatic and hydrophobic (the lignin pathway predominates), while peat HSs are more aliphatic and more highly enriched with oxygen, which may indicate a greater contribution from the products of microbial metabolism and polycondensation reactions [6].

**Figure 1:**
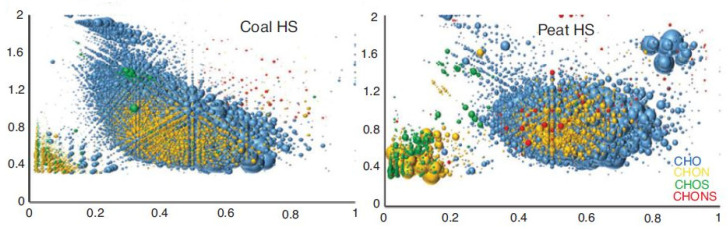
Chemical space of humic substances of coal (Coal HS) and peat (Peat HS), presented on the 2D Van Krevelen diagram, reproduced with permission from [6], 2019, De Gruyter.

As noted earlier, based on the results of studying the structure of HA using the FTICR MS method, several tens of thousands of molecular formulas were identified in the HA structure [6,8,9,10], which became the basis for such a concept as the “chemical molecular space of HS” emerged, which encompasses a very significant portion of the combinatorial space of all possible molecules with the atomic composition CHO. Examples of such chemical molecular spaces for HA of coal and peat shown in Figure 1.

The major components of HA are phenolic, enolic and quinone fragments, carboxyl and ether functional groups, carbohydrate, lipid and peptide structures are represented to a lesser extent [11]. The HA macromolecule consists of hydrophilic fragments consisting of –OH groups and hydrophobic fragments consisting of aliphatic chains and aromatic rings. Phenolic and carboxyl groups are responsible for the weakly acidic behavior of HA. The total acidity (acidity due to phenolic and carboxyl groups) of compounds isolated from soil, water, and geological sediments is approximately 6 meq/g [11].

**Figure 2:**
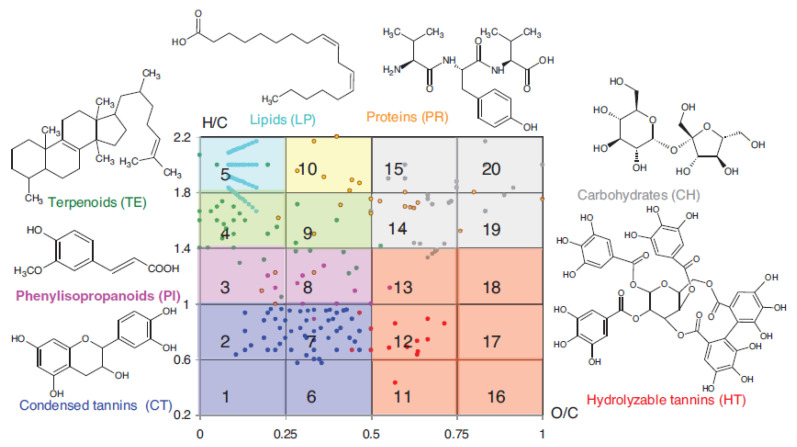
Mapping the van Krevelen diagram of the humic acids by classes of bioorganic compounds, reproduced with permission from [6], 2019, De Gruyter.

Mapping such Van Kevelen diagrams (Figure 2) allows classifying the main components of HS into classes of organic compounds [6].

Thus, HSs are characterized by the presence of a large number of different functional groups and stable radicals, which ensures their ability to form a variety of intra- and intermolecular bonds that determine their redox, chelating, and protolytic properties, as well as their participation in ligand exchange and heterogeneous processes. All of these effects are key to their biological activity and determine their unique chemical and pharmacological properties.

## 2. Pharmacological Effects of Humic Substances and Their Signaling Mechanisms

The largest amounts of HS accumulate in natural objects such as coal, peat, sapropel, and mumie, which have been used for over 3000 years in traditional medicine and veterinary science as immunomodulators, biogenic stimulants, adaptogens, stress protectors, anti-inflammatory and antitumor agents, antioxidants and antihypoxants, hepatoprotectors and cardioprotectors, enterosorbents, antimicrobial drugs, etc. Despite the large number of literary sources containing data on the high and multidisciplinary pharmacological activity of HS, it is still possible to combine all their biological effects into several broad groups.

### 2.1. Immunotropic Action

*Immunological significance of humic substances*

Discoveries made in the first decade of the 21st century—several of which were awarded Nobel Prizes—demonstrated that the immune system participates not only in the development of immunopathological conditions (allergies, autoimmune diseases, immunodeficiencies, graft-versus-host responses, and others) but also in bacterial infections and cancer. Thus, normal immune function is essential for maintaining both physical and mental health. Consequently, expanding the range of therapeutic agents capable of modulating immune homeostasis is a key task in all fields of medicine. Humic substances (HS) are also considered to exert measurable effects on multiple components of the immune system.

Numerous studies report that HS influence humoral and cellular immunity [12,13,14,15,16,17,18,19,20] and many others, increase the phagocytic activity of leukocytes and lysozyme [21,22], enhance the phagocytic activity of neutrophils [12] and their adhesion to the endothelium [23], modulate transcription factor expression [24], stimulate activated lymphocyte proliferation [17], regulate the synthesis of pro- and anti-inflammatory cytokines [12,17,19], protect cells from apoptosis [25], inhibit the growth of breast cancer cells in the presence of glucan [14], exert a positive effect on the complement system [19,23], increase the number and functional activity of macrophages, neutrophils, and cytotoxic T cells [14,15,16,26]. A study [13] describes pleiotropic effects of HS that depend on their structural features, including suppression of metastasis formation in the absence of chemotherapeutic agents (using Lewis lung carcinoma as a model), stimulation of IL-2 secretion by mouse splenocytes in vitro, induction of ovalbumin-specific antibodies, reduction in splenic cell apoptosis during early stages of immune activation, acceleration of wound healing in vitro using HaCaT keratinocytes, and protection from LPS-induced hepatotoxicity. The authors [13] conclude that the carbohydrate component of the HS macromolecule plays a key role in mediating their immunotropic activity—analogous to the mechanism of action of glucan.

*Modulation of cell adhesion and inflammatory responses (preclinical studies in vitro)*

Being surface-active compounds, humic substances (HS) can influence cellular adhesion and thereby exert anti-inflammatory effects. In vitro experiments demonstrated that potassium humate derived from brown coal dose-dependently inhibited the expression of the CR3 receptor in neutrophils activated with phorbol-12-myristate-13-acetate (PMA). It also inhibited the adhesion of PMA-stimulated neutrophils to the BHK331-7 hamster kidney cell line expressing the adhesion molecule ICAM-1 (a CR3 ligand). Additionally, potassium humate suppressed the release of myeloperoxidase and eosinophil cationic protein from activated neutrophils and eosinophils, which may contribute to the anti-inflammatory effects of HS [23].

A study by R.J. Gau et al. demonstrated that pretreatment of human umbilical vein endothelial cells (HUVECs) with commercial HA (Aldrich, St. Louis, MO, USA) inhibited LPS-induced expression of adhesion molecules including ICAM-1, VCAM-1, and E-selectin, as well as NF-κB activation [27]. Conversely, C.H. Chen et al. showed that HA from the same commercial source (Aldrich, USA) enhanced neutrophil adhesion to HUVEC culture and increased oxidant production in a dose-dependent manner. The ability of HA to enhance adhesion was mediated through the activation of ERK, mitogen-activated protein kinase p38 (p38 MAPK), phosphatidylinositol-3-kinase (PI3K) and NF-κB pathways in neutrophils [28]. Despite using HA from the same manufacturer, the opposite effects reported in the two studies can be explained by the high heterogeneity of HS and the cell-specific nature of their activity. Humic acids represent a complex mixture of numerous organic components, and even minor differences in the distribution of molecular fractions or the presence of trace impurities between batches can shift their biological effects toward either pro- or anti-inflammatory responses. In addition, the activity of HA strongly depends on the cell type and the signaling pathways they engage. In endothelial cells, they may suppress LPS-induced expression of adhesion molecules and NF-κB activation, whereas in neutrophils they can activate ERK, p38 MAPK, and PI3K and enhance adhesion. Differences in concentrations and sample preparation conditions may further contribute to variability in the observed responses. Thus, the reported discrepancies reflect the pleiotropic nature of HA and their sensitivity to cellular context and experimental conditions.

*Regulation of transcription factors and intracellular signaling pathways (preclinical studies in vitro)*

The modulation of transcription factor expression and intracellular signaling cascades by humic substances (HS) has been reported in several studies [24,29,30]. In the work of Y.C. Hseu et al. [24], synthetic humic acid (HA) was shown to alter macrophage activation, particularly affecting the production of pro-inflammatory mediators and the activation of transcription factors, using the RAW 264.7 murine macrophage cell line as a model. HA increased the expression of NF-κB and activator protein-1 (AP-1), followed by induction of inducible nitric oxide synthase (iNOS) and cyclooxygenase-2 (COX-2), resulting in enhanced production of NO and prostaglandin E_2_. Exposure to HA also led to increased formation of reactive oxygen species (ROS) and nitrotyrosine, as well as activation of the Akt and MAPK signaling pathways. Notably, NF-κB activation was mediated by ROS and Akt, whereas AP-1 activation was mediated by JNK and ERK. In addition, intraperitoneal administration of HA to mice elevated serum levels of TNF-α and IL-1β [24].

Studies by E.S. Trofimova et al. [29,30] further demonstrated that peat-derived HA stimulate nitric oxide production in cultured mouse peritoneal macrophages. This effect was mediated through activation of Toll-like receptors TLR-2 and TLR-4 and involved intracellular signaling components, including p38 MAPK, PI3K, MEK1/2, cAMP, NF-κB [30], the IKK-2 kinase complex, and the NF-κB transcription factor [29].

**Figure 3:**
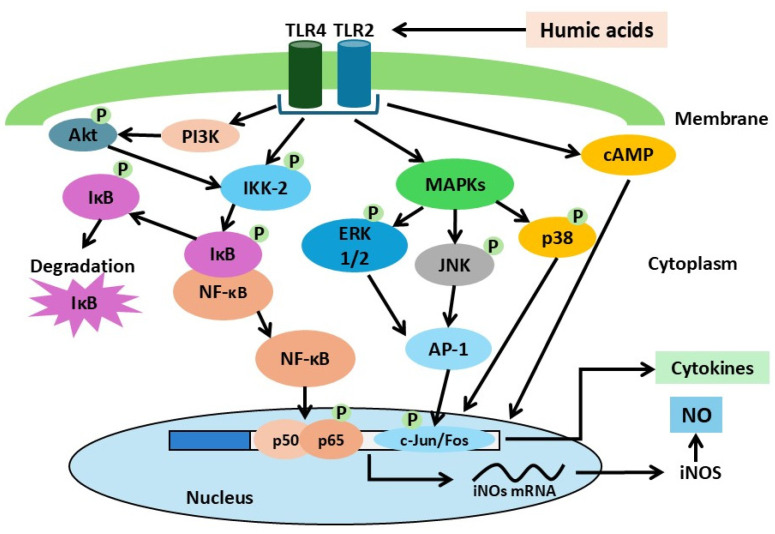
Hypothetical model of macrophage activation by humic acids. Pro-inflammatory activation of macrophages is proposed to occur through engagement of TLR-2 and TLR-4 membrane receptors, followed by activation of MAPK signaling pathways (p38, ERK, JNK), PI3K/Akt, the IKK-2 kinase complex, and cAMP, ultimately leading to stimulation of the transcription factors NF-κB and c-Jun.

The available data on HA-activated signaling cascades can be integrated into a general model (Figure 3).

*Effects of humic substances on innate and adaptive immunity (preclinical studies in vivo)*

In the study of R. Habibian et al. [20], a veterinary preparation containing humic acids (HA) enhanced the humoral immune response against *Brucella melitensis* and increased the phagocytic activity of mononuclear cells in rats in a dose-dependent manner [20]. An enhanced humoral immune response, characterized by a dose-dependent increase in ovalbumin-specific antibody titers, was also observed in rats fed HA and FA isolated from brown coal. Histological examination revealed enlargement of B-dependent regions (the marginal zone) and increased germinal center diameter in the spleen, as well as enlargement of the germinal centers in lymphoid follicles of the ileum in animals receiving HA and FA as dietary supplements [31].

A dietary supplement based on HA improved the humoral immune response in broiler chickens by increasing antibody titers against infectious bronchitis virus and Newcastle disease virus without altering key biochemical blood parameters [32]. In addition, HA enhanced innate cellular immunity by increasing phagocytic activity, and modulated adaptive cellular immunity by increasing the proportion of CD4+ lymphocytes while decreasing CD8+ lymphocytes [18]. It has been established that supplementation with HS also increased the proportion of T helper cells (CD4^+^CD8^−^) in piglets by approximately 1.5-fold [33].

In mice immunized with ovalbumin, HS from various sources demonstrated adjuvant properties against the antigen by enhancing specific antibody production. In monotherapy, HS also stimulated the uptake of synthetic particles by murine peripheral blood neutrophils and peritoneal macrophages [12].

*Influence of humic substances on cytokine production (preclinical studies in vitro and in vivo)*

Numerous studies have investigated the effect of humic substances (HS) on the production of pro- and anti-inflammatory cytokines by immunocompetent cells.

Potassium humate derived from brown coal significantly inhibited at a dose of 40 μg/mL the in vitro release of pro-inflammatory cytokines TNF-α, IL-1β, and IL-6 from phytohemagglutinin A (PHA)-stimulated mononuclear lymphocytes, and suppressed activation of both the alternative and classical complement pathways [17]. Potassium humate also dose-dependently increased lymphocyte proliferation stimulated by PHA and by pokeweed Mitogen (PWM). The authors attributed the increased proliferation to enhanced IL-2 production, consistent with the findings of G.K. Jooné et al. [23], who demonstrated that oxyhumate increased lymphocyte proliferation not only in vitro but also ex vivo following administration of a non-toxic dose to HIV-positive individuals. Oxyhumate also enhanced IL-2 receptor expression in vitro while reducing IL-10 production.

M. Verrillo et al. [34] demonstrated the anti-inflammatory effects of humic acids isolated from lignite and composted artichoke residues. Humic acids reduced the expression of IL-6 and IL-1β genes in human keratinocyte (HaCaT) cells. Furthermore, trypan blue staining revealed that HA increased the viability of keratinocytes pre-exposed to urban dust. The authors proposed that these bioactive properties of HA arise from the hydrophobic characteristics of HA, which promote their adhesion to the surface of target cells, and from the conformational stability of humic acids supramolecular associations. Less stable conformations of HA may promote the release of bioactive components upon cell interaction, thereby reducing inflammatory cytokine expression [34].

In a study [2], coal-derived HS modulated the balance between nitric oxide (NO) and arginine metabolism in murine peritoneal macrophages. At the most effective concentration (10 μg/mL), HS enhanced a Th1-type immune response, resulting in the formation of effector cells for combating intracellular pathogens (viruses and intracellular bacteria), such as cytotoxic T-lymphocytes, which also confer anti-tumor immunity. Furthermore, coal-derived HS also increased the production of pro-inflammatory cytokines by murine macrophages and lymphocytes. Coal-derived HS also increased the production of pro-inflammatory cytokines by murine macrophages and lymphocytes. Lipopolysaccharide (LPS) was used as a standard macrophage activator, and splenocytes from mice were treated with the T-cell mitogen concanavalin A (ConA) to stimulate the T-lymphocyte pool. The results showed that HS significantly enhanced the spontaneous production of key pro-inflammatory cytokines TNF-α (by 10.9-times) and IL-1β (by 7.2-times) relative to untreated cells. Incubation of ConA-stimulated splenocytes with HS led to a 2.4-fold increase in their production of the pro-inflammatory cytokine IL-2 compared to the control. The authors [2] concluded that the investigated sample of coal HS is promising candidates for the development of effective immunotropic drugs targeting Th1-dependent immune response deficiency, such as chronic, persistent, and recurrent infectious diseases, as well as cancer.

In a study of M.H. Şehitoğlu et al. [35], HA demonstrated protective anti-inflammatory effects in experimental gastric ulceration. Treatment of experimental ulcers in Wistar rats with HA suppressed iNOS activity in gastric tissue (0.16 ± 0.40 vs. 0.66 ± 0.81 in control), thereby reducing inflammation and preventing damage to the gastric mucosa, as well as reducing the number of apoptotic cells. HA treatment also lowered expression of pro-inflammatory cytokines IL-6, TNF-α and caspase-3 in stomach tissue cells while the level of the anti-inflammatory cytokine IL-10 was increased in the HA-treated groups [35].

The anti-inflammatory properties of HS may be useful not only in the treatment of immune-mediated disorders, but also in managing other pathologies associated with low-grade chronic inflammation. In the study [36] human umbilical vein endothelial cells (HUVEC) cultured under experimental hyperglycemic conditions were treated with humic water (water derived from underground sources containing HA). Treatment reduced the production of pro-inflammatory cytokines TNF-α and IL-6, increased cell proliferation and inhibited the activation of key signaling pathways—MAPK (mitogen-activated protein kinases), NF-κB (nuclear factor κB) and STAT3. Based on these findings, the authors suggest the potential use of humic water as supportive therapy for endothelial dysfunction in diabetes mellitus.

Another study [37] also reported that an alginate–HA-based biomaterial produced by Sigma (USA) suppressed the production of the proinflammatory cytokine IL-1β in the human monocytic cell line THP-1 without exerting cytotoxic effects and did not alter TNF-α production.

In the study by M. Verrillo et al. [38] HS isolated from composted fennel residues exhibited antioxidant and anti-inflammatory properties in human gastric adenocarcinoma (AGS) cells stimulated with *Helicobacter pylori* culture filtrate. The test substances reduced the production of IL-12, IL-17, and G-CSF by AGS cells.

In an in vitro model, HS extracted from chalk shale inhibited the activity of the enzymes COX-2 and 5-LOX [39]. These enzymes are pro-inflammatory enzymes, play key roles in arachidonic acid metabolism, and are involved in the production of eicosanoids, including thromboxane, leukotrienes and prostaglandins, which drive inflammatory responses [40]. Accordingly, HA and FA may limit the formation of inflammatory mediators in cells.

V. Vetvicka et al. [12] demonstrated that repeated administration of HA from various sources stimulated IL-2 production by mouse splenocytes. In addition, elevated levels of both pro- and anti-inflammatory cytokines (IL-2, IL-4, IL-5, IL-6, TNF-α, and monocyte chemoattractant protein-1 (MCP-1)) were detected in the blood of treated animals.

In study [41] fulvic acids (FA) derived from mumie were shown to enhance the production of reactive oxygen species (ROS) and nitric oxide (NO) in murine peritoneal macrophages. These mediators are essential for pathogen elimination through ROS-dependent signaling and for bactericidal activity that activates leukocyte immune responses. The balance between ROS production and antioxidant (AO) defenses is critical for maintaining cellular redox homeostasis, which is important for DNA synthesis, gene expression, and enzymatic activity. Similar results were reported by R.G.P.T. Jayasooriya et al. [42], who evaluated the immunomodulatory capacity of FA in RAW 264.7 macrophages (NO production). FA increased the expression of inducible nitric oxide synthase (iNOS) protein and mRNA and enhanced the DNA-binding activity of the nuclear transcription factor NF-κB. The authors [42] concluded that FA activate NF-κB signaling thereby inducing iNOS mRNA expression and NO synthesis in RAW 264.7 cells.

E.S. Trofimova, M.V. Zykova and co-authors [43,44,45] demonstrated that peat-derived HA stimulated the secretion of both pro- and anti-inflammatory cytokines by human peripheral blood mononuclear cells, murine splenocytes and peritoneal macrophages. However, in the presence of mitogens, HA increased the production of anti-inflammatory cytokines in the lymphocyte fraction of mononuclear cells while enhancing the production of pro-inflammatory cytokines by macrophages [43,44,45], indicating the immunoregulatory properties of HA under inflammatory conditions.

*Predictive modeling of the immunotropic activity of humic acids (a study in cell culture of peritoneal macrophages)*

A fundamental study [46] describes a novel method for evaluating the biological activity of peat-derived HA, using an artificial neural network (ANN) to process spectroscopic measurements in the infrared and visible ranges. This approach is based on the quantitative structure-activity relationship (QSAR) concept and employs a multilayer perceptron (MLP) model to improve predictive efficiency. The developed MLP model enabled the estimation of the biological activity for full vertical peat cores collected from an oligotrophic peat bog located in the southern taiga zone of Western Siberia (northeastern spurs of the Great Vasyugan Mire). A total of 42 samples were collected from the cores. The research protocol included spectroscopic analysis (in the infrared and visible ranges) and a biological assay using activated peritoneal macrophages as a reference method for direct measurement of HA biological activity. Numerical experiments confirmed the consistency between measured and estimated biological activity, with a coefficient of determination R^2^ = 0.97. These experiments also demonstrated that the MLP model significantly outperforms traditional linear multiple regression models, primarily due to the substantial nonlinearity of the structure-activity relationships. The study revealed that the biological activity of peat HA can be estimated using an ANN model trained on electronic and IR spectra. The primary advantages of the proposed method are its simplicity and speed. Furthermore, it can be implemented using sufficiently common and accessible laboratory equipment. Another positive aspect is the flexibility of the developed MLP model, which can be easily retrained and adapted to other HS.

*Anti-Allergic and Anti-Inflammatory Properties (preclinical studies in vitro and in vivo)*

In the study by H. Motojima et al. [47], the effect of FA on immediate-type allergic reactions and the underlying mechanisms of action were examined in KU812 basophil cells activated with phorbol 12-myristate 13-acetate and the calcium ionophore A23187. The inhibitory action of FA on degranulation in ionophore-stimulated KU812 basophil cells was assessed by measuring histamine release which was reduced at FA concentrations of 0.1–10.0 µg/mL. The mechanism of cell degranulation suppression was studied using DNA microarray analysis to identify genes differentially expressed in response to FA in ionophore-stimulated KU812 cells. Of the 201 genes represented on the array, 28 showed increased expression, 173 showed decreased expression, and the expression of 71 genes changed more than twofold following 15 min of FA pretreatment; 16 genes were selectively suppressed. Based on these findings, the authors concluded that FA influence the expression of genes involved in signal transduction, cytokine-cytokine receptor interaction, immune response, cell adhesion molecules, and the IgE β-subunit receptor response [47].

The study [48] also showed that FA can reduce the release of histamine and β-hexosaminidase from IgE-sensitized mast and basophil cells. Treatment with FA at a dose of 200 µg/mL, decreased TNF-α expression following lipopolysaccharide stimulation in differentiated human monocytes (U937), and similarly reduced cyclooxygenase-2 and prostaglandin E2 secretion following homocysteine stimulation in primary human monocytes.

In a rat paw edema model, it was found that the pro-inflammatory or anti-inflammatory effects of HA depend on their dose [49]. The anti-inflammatory mechanism was associated with the inhibitory effect of 5-lipoxygenase, while the pro-inflammatory mechanism was associated with the release of neutrophil granulocytes. At low concentrations (10–80 μg/mL), HA increased TNF-α production in LPS-stimulated cells of the human myeloid leukemia promonocyte line U937 by threefold (pro-inflammatory activity), whereas at higher concentrations (>100 μg/mL), HA reduced TNF-α production by 10-fold (anti-inflammatory activity) without inducing cytotoxicity. HA did not stimulate TNF-α release in the absence of LPS, indicating that HA are not inflammatory agents.

The pronounced anti-inflammatory effects of HS have been demonstrated in several animal models of inflammation. In a contact hypersensitivity model induced by 2,4-dinitrofluorobenzene (2,4-DNFB), humate derived from brown coal exerted significant anti-inflammatory activity [50]. The humate was administered intragastrically to rats for eight days immediately following the sensitization phase. The 2,4-DNFB-induced contact hypersensitivity reaction represents an in vivo model of delayed-type hypersensitivity (DTH) and mimics contact dermatitis in humans [50]. Potassium humate reduced ear swelling to the same extent as prednisolone [51]. The study [51] similarly demonstrated that orally administered HS (60 mg/kg) inhibited DTH reactions, reduced carrageenan-induced edema, and suppressed transplant rejection in rats immunized with sheep erythrocytes. These findings are consistent with the anti-inflammatory effects of humic and fulvic acids observed in a rat skin wound model, where a reduction in inflammatory cell infiltration at the site of injury was reported [52].

P.J.W. Naudé et al. [53] investigated the effects of potassium humate administered orally on the delayed-type hypersensitivity (DTH) response induced by sheep red blood cells, carrageenan-induced nonspecific inflammation, and allogeneic graft-versus-host disease (GVHD) in rats. Potassium humate had no effect on the DTH response but significantly suppressed carrageenan-induced edema. Furthermore, potassium humate inhibited GVHD in normal and immunodeficient rats. In a model of carrageenan-induced edema, HS extracted from chalk shale significantly (33, 47 and 54% at 50, 100 and 200 mg/kg dose, respectively) reduced paw edema in mice [39]. As demonstrated in [54], FA suppressed carrageenan-induced inflammation, as effectively as the non-selective COX inhibitor indomethacin. Moreover, unlike indomethacin, FA did not cause the development of systemic side effects, particularly ulcerogenesis.

Humic substances are capable of both inhibiting the secretory degranulation of phagocytes and activating their synthetic activity, as well as promoting their chemotaxis to sites of allergic inflammation [17]. In a study [48] HSs are considered as promising compounds for anti-cytokine therapy in a number of autoimmune and inflammatory diseases, since they reduce TNF-α expression in LPS-differentiated U937 monocytes. Moreover, HS suppress LPS-induced expression of adhesion proteins on the cell surface and reduce activation of nuclear factor NF-κB in human umbilical vein endothelial cells (HUVECs), thereby modulating their inflammatory response [55].

The antiallergic effect of peat-derived HA has been demonstrated in models of delayed and immediate hypersensitivity in animals. Repeated administration of various HA preparations to animals suppressed DTH induced by sheep red blood cells [44] and reduced the severity of the general anaphylactic reaction following ovalbumin sensitization, as well as the production of IgE and G1 and the degree of mast cell degranulation [56].

**Figure 4:**
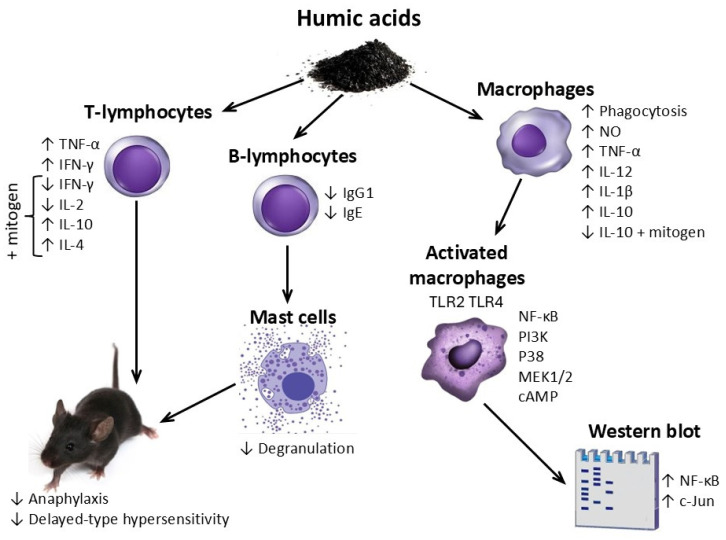
General scheme of the immunotropic effects of humic substances.

The above mentioned immunotropic effects of HSs are schematically represented in Figure 4.

HSs are believed to influence the lymphocyte component of the immune system (T and B lymphocytes) and antigen-presenting cells (macrophages), and to suppress allergic reactions and inflammation at the organismal level.

*Clinical evidence of immunomodulatory and anti-Inflammatory activity*

In addition to experimental data obtained using cell cultures and animal models, there are reports of clinical studies describing the immunotropic and anti-inflammatory effects of HS. For example, a significant increase (*p* < 0.05) in PHA-stimulated proliferation of mononuclear leukocytes was observed ex vivo in HIV-infected individuals after the administration of 4 g of oxyhumate per day for 2 weeks compared to the group receiving placebo [57].

Convincing results were obtained in two randomized, double-blind, placebo-controlled clinical trials. In the first study, potassium humate was administered orally to patients with allergic rhinitis [58], and in the second, to patients with knee osteoarthritis [19,59]. In the allergic rhinitis trial, potassium humate reduced cutaneous reactions to an allergen, while in the osteoarthritis trial it improved patients’ physical condition and reduced the level of C-reactive protein in the blood.

In a double-blind crossover study, the anti-inflammatory effect of HS was also observed when applied as a 4.5% cream to volunteers with allergies who underwent an intradermal antigen test. Oxifulvic acid inhibited the development of an inflammatory reaction 15 min after application, showing efficacy comparable to 1% hydrocortisone cream. The researchers hypothesize that the mechanism of action is related to antioxidant properties and suppression of IL-2 production [60].

The anti-inflammatory effect of HS derived from sulfide muds was demonstrated in the study [61] using a model of experimental adjuvant polyarthritis. A reduction in autoimmune activity (decreased ESR, leukocytosis, myeloperoxidase, fibronectin, IL-1, and TNF-α), normalization of the properties of monocyte-macrophage system and mitigation of the imbalance in immunoregulatory T-lymphocyte subsets were observed.

Research concerning the use of peat extracts in clinical practice developed in Poland and led to the creation of Tołpa Peat Preparation (TPP). TPP was shown to exert an interferonogenic effect (induced the production of endogenous IFN-α and IFN-γ), enhance the cytotoxicity of tumor necrosis factor (TNF-α) in human peripheral blood leukocytes, and increase serum immunoglobulin levels, primarily IgM and IgG, and the acute phase protein alpha1-antitrypsin. Due to its ability to enhance the immune response, TPP has found application in sports medicine as an oral immunomodulator [62].

The intravenous administration of TPP (5 mg/kg) to rabbits increased the percentage of phagocytic cells and enhanced the phagocytic activity of neutrophils. Meanwhile, against the background of LPS-induced fever, TPP at a dose of 50 mg/kg completely inhibited the development of endotoxic shock [63]. The same drug (TPP) exhibits immunocorrective properties against the background of antibacterial therapy with a number of antibiotics (ampicillin, amikacin, doxycycline, rifampicin, rifamycin), and also contributes to significant (*p* < 0.001) localization of inflammation and enhanced vasculogenesis during xenotransplantation [64]. Tołpa Peat Preparation is capable of stimulating angiogenesis, including that induced by human leukocytes, and reducing spontaneous IL-1 production by monocytes in patients with rheumatoid arthritis [65,66]. This drug also accelerated the healing of gastric ulcers caused by ethyl alcohol [67]. However, in their review, J. Drobnik and A. Stebel point to significant shortcomings in obtaining clinical evidence of the effectiveness of TPP and indirectly recommend only its external use [68]. Currently, TPP is not used as a medicinal product. However, many cosmetic products containing peat are available for sale in Poland (dental gels, ointments, toothpastes, creams, bath emulsions, shampoos, etc.).

Thus, the mechanism of action of HS can be mediated by the activation of both classical and alternative complement pathways, as well as phagocyte degranulation and the production of various anti- and pro-inflammatory cytokines.

### 2.2. Antibacterial Action (Preclinical Studies In Vitro and In Vivo)

Infectious diseases, due to their high contagiousness, the ability of pathogens to remain viable for long periods in the environmental objects, the high intensity of migration processes, the ever-increasing level of urbanization and population density, as well as the development of protective mechanisms in pathogens against drugs, continue to hold a leading position in the structure of human morbidity. This problem is further exacerbated by the uncontrolled use of antibiotics and antiviral agents in medicine and many sectors of economic activity, as well as a sharp reduction in the development and production of new drugs by pharmaceutical companies aimed at combating pathogens of nosocomial and viral infections. Therefore, the search for molecules, preferably of natural origin, with antimicrobial and immunomodulatory effects and incapable of provoking the development of drug resistance is relevant. Thus, against the backdrop of the progressive growth of multi-resistant microorganisms in recent years, there has been a growing interest in HS obtained from various sources as potential natural preparations that increase the sensitivity of bacteria to antibiotics [69]. Humic substances have found application along with prebiotics and probiotics as an alternative to antibacterial therapy [22]. The bactericidal and fungicidal activity of HA is associated with the presence of phenols, carboxylic acids and quinones in their structure [70]. The relationship between phenolic compounds and antimicrobial activity against human pathogens was reported in studies by C. Cueva et al. [71] and L. Bouarab-Chibane et al. [72]; however, the mechanism of this activity is not fully understood. It is assumed that the interaction of phenols with the active centers of various enzymes can cause irreversible changes in the permeability of the cell membrane or its integrity, followed by the death of the bacterial cell [73]. The authors suggest that the different sensitivity of Gram-positive and Gram-negative bacteria to polyphenols is associated with the more complex molecular structure of the cell membrane of Gram-negative bacteria, which slows down the passage of chemicals into the cell [69].

In the work of G. Kupryszewski et al. [74] the antimicrobial activity of twelve preparations of HS (HA and FA) isolated from seawater, marine bottom sediments and lake water was studied. The authors [74] found that the drugs inhibited the growth of 11 strains of anaerobic bacteria (*Bacteroides fragilis* ATCC 25285, *Bacteroides vulgatus* ATCC 8482, *Bacteroides ovatus* ATCC 8483, *Fusobacterium nucleatum* ATCC 25585, *Peptostreptococcus anaerobicus* ATCC 27337, *Propionibacterium acnes* ATCC 11827, *Clostridium perfrigens* ATCC 13124*, Peptostreptococcus productus*, *Actinomyces bovis*, *Clostridium difficile*, *Clostridium septicum*), 8 strains of aerobic bacteria (*Enterococcus faecalis* ATCC 29212, *Staphylococcus aureus* ATCC 25923, *Bacillus cerus* ATCC 10876, *Bacillus subtilis* NTCT 8236, *Escherichia coli* ATCC 25922, *Klebsiella pneumoniae* ATCC 13883, *Pseudomonas aeruginosa* ATCC 27853, *Acinetobacter baumannii* ATCC 19606) and 2 strains of yeast-like fungi (*Candida albicans*, *Candida glabrata*) [74].

Humic substances (HA and FA) extracted from chalk shales showed significant antibacterial activity in vitro against *Salmonella typhi* (minimum inhibitory concentration (MIC)—0.82 mg/mL), *Pseudomonas aeruginosa* (MIC—0.87 mg/mL) and *Escherichia coli* (MIC—0.79 mg/mL)*, Bacillus subtilis* (MIC—0.93 mg/mL)*, Staphylococcus aureus* (MIC—1.12 mg/mL) and antifungal activity against *Alternaria alternata* and *Fusarium solani* (MIC—0.60 and 0.68 mg/mL, respectively) [39].

Using the diffusion disk method, M. Verrillo et al. [69] revealed antimicrobial activity of HS isolated from composted agricultural biomass against such Gram-positive bacteria as *Staphylococcus aureus* and *Enterococcus faecalis*. Less sensitivity was found for Gram-negative bacterial strains such as *Escherichia coli* and *Klebsiella pneumoniae*. The authors [69] associate the antimicrobial properties of the studied HS with their specific molecular composition and the conformational stability of their superstructures. In particular, the samples with the highest content of hydrophobic aromatic and phenolic components and with a more rigid conformational organization had the greatest antibacterial properties [69]. Similar effects of HS in relation to both gram(+) and gram(−) bacteria, some types of fungi, in particular, *Staphylococcus aureus*, *Escherichia coli*, *Pseudomonas aeruginosa*, *Neisseria gonorrhoeae*, *Klebsiella pneumonia*, *Candida albicans* and others were shown in the work by Skliar et al. [75]. It is also reported that the most probable mechanisms of the antibacterial action of HS are their ability to disrupt the metabolism of essential nutrients in microbial cells, as well as to form interionic bonds with polyhydroxyalkanoates of microorganisms and subsequent changes in their biological properties [76].

In recent decades, the problem of antimicrobial resistance has ceased to be a purely medical issue. Environmentalists are increasingly raising this issue and attempting to resolve it, concerned that nearly 80–90% of antibiotics consumed are excreted unchanged by humans and animals and contribute to further pollution of water in treatment plants, runoff from agricultural lands, livestock facilities, etc. When present in the environment, antibiotics contribute to the development of bacterial resistance to them, as well as the transmission of their resistance genes to future generations. Based on experimental data showing that binding of macrolide, tetracycline, fluoroquinolone antibiotics and sulfonyl amide drugs with HS leads to a decrease in their mobility, reactivity and bioavailability, HS were proposed as effective sorbents that purify aquatic and soil ecosystems and prevent the spread of antibiotic resistance genes in the most common causative agents of nosocomial infections of Gram-negative bacteria [77].

Fulvic acids have also been described as having potential use as an antimicrobial agent, including for the treatment of purulent wounds [78], 8 strains (*Streptococcus faecalis*, *Staphilococcus aureus*, *Pseudomonas aeruginosa*, *Escherichia coli*, *Streptococcus pyogenes*, *Klebsiella pneumonia*, *Proteus mirabilis*, *Candida albicans*) were tested. All strains were susceptible to oxifulvic acid at a concentration of 15 g/L, while *Enterococcus faecalis* and *Klebsiella pneumoniae* exhibited susceptibility at concentrations up to 5 g/L.

The antibacterial and antifungal properties of FA are further described in the patent by J. Dekker et al. [79]. In vivo studies were conducted on several known pathogens using a 25.4% FA solution and a 4.5% FA-based cream (β*-Hemolytic streptococcus*, *Streptococcus faecalis*, *Klebsiella pneumonia*, *Pseudomonas aeruginosa*, *Candida* spp., *Escherichia coli*, *Proteus mirabilis*, *Staphylococcus aureus*). The authors [79] noted the presence of bactericidal and/or bacteriostatic activity of varying degrees against certain microorganisms, including in a cream formulation. Furthermore, FA can exhibit bactericidal and bacteriostatic properties not only as monotherapy but also in combination therapy, for instance, with colistin, meropenem, oxacillin, gentamicin, fluconazole, or amphotericin B [80,81,82], this demonstrates the synergistic effect of FA in combination with antibacterial agents. It was noted that FA in combination with fluconazole and amphotericin demonstrated high efficacy against fluconazole- and amphotericin-resistant *Candida* spp. Fulvic acids may serve as an effective and safe agent for the treatment of various diseases of bacterial, viral, and fungal etiology.

Thus, HS from various sources have a wide range of antibacterial and antifungal activity, which is due to the peculiarities of their chemical structure.

### 2.3. Antiviral Action (Preclinical Studies In Vitro)

Rapid evolution and mutation of viruses leads to their resistance to antiviral drugs and, as a consequence, there is a need for new antiviral agents suitable for the treatment of resistant infections [83,84,85,86].

Humic substances have long been known for their antiviral activity. Thus, oxyhumates isolated from bituminous coal, under conditions of reduced IL-10 production, inhibited infection of human MT-2 lymphoblastic cell culture with *HIV-1* [57,87], which led to increased synthesis of IL-2 by CD4+ lymphocytes, as well as the expression of receptors for this cytokine on their surface. Furthermore, blockade of viral replication was observed due to inactivation of the viral particle and inhibition of syncytium formation in cell cultures. The authors [57,87] attribute the inhibition mechanism to the effect of oxyhumates on the V3 loop and partial influence on the CD4 binding site of the viral envelope protein gp120, i.e., the virion’s surface glycoprotein with the target cell receptor. Since the V3 loop of gp120 contains a net positive charge in lymphocytotropic HIV isolates, negatively charged oxyhumates can specifically bind to this site. Moreover, no viral resistance to oxyhumates was observed throughout the 12-week in vitro experiment, unlike with single-target HIV inhibitors [57,87]. The study authors believe that oxyhumates are promising molecules that can be included in treatment regimens for immunocompromised patients [57,87].

The study [88] established that HS possess antiviral activity against members of the Herpesviridae family (*HSV-1*, *HSV-2*, and *HCMV*) and *Paramyxoviridae* (*RSV*), i.e., unlike many chemotherapeutic agents, they have a fairly broad spectrum of action. Moreover, the ability of HS to inhibit viruses manifests itself at an early stage of their replicative cycle, i.e., at the penetration stage.

The combination of virucidal activity against *HIV-1*, hyperbranched structure, enrichment of molecules with several active centers in the form of carboxyl and hydroxyl groups, and low toxicity are considered by Y.V. Zhernov et al. [89] as an opportunity to create complex dendrimer-like microbicides based on HS. Also Y.V. Zhernov et al. [90] studied in vitro the antiviral activity against *HIV-1* of a set of HS, including HA from coal, peat and peloids, as well as FA and hymatomelanic acids. All humic materials used in this study exhibited antiviral activity, with HA having the greatest antiviral effect among all the substances studied. The authors associate the more pronounced antiviral activity of HA with a high lipophilicity index, i.e., the ratio of aromatic to aliphatic carbon, which may indicate the leading role of aromatic structures in interaction with HIV. Aromatic structures with alkyl substituents, terpenoids, N-containing analogs of typical flavonoids, and aza-podophyllotoxins were identified as potential carriers of antiviral activity. The authors [89] suggest that hydrophobic humic materials interfere with fusion and inhibit the activity of *HIV-1* reverse transcriptase, as has been experimentally confirmed.

There is also a patent for the treatment of HIV and AIDS using HA [91], where anti-HIV-1, anti-syncytial and IL-2 immunostimulating effects have been proven. According to studies, HS inhibits *HIV-1* viral replication in human lymphocytes and their infectivity, blocking syncytium formation between infected and uninfected lymphocytes. The recommended therapeutic dose is 7.5 mg of HA per 1 kg body weight.

Using models of MT-4 lymphoblastoid cells infected with *HIV-1* strains and *Vero cells* infected with *herpes simplex virus type 1* (*HSV-1*), D.N. Nosik et al. [92] studied the antiviral activity of HS from brown coals of the Kansk-Achinsk deposit. It has been established that HSs have antiviral activity against both the RNA-containing *HIV-1* virus and the DNA-containing *HSV-1* virus, which indicates that the type of nucleic acid in the virus does not play a fundamental role in the antiviral action of these substances.

It is also noted that HA-like polymers obtained by oxidation of ortho-diphenolic compounds have tropism towards *HSV-1* [93]. Functional analysis of the obtained compounds showed that the presence of carboxyl groups and phenolic hydroxyls, as well as conjugated multiple bonds in the side chains, enhances the virucidal activity and reduces the cytotoxicity of the studied polymers [93]. In the study [94] virus-neutralizing activity of HS against *Tick-borne encephalitis virus* (*TBEV*) was found as a result of the interaction of HS with positively charged proteins of the viral envelope. This confirmed once again that HS, like other polyanionic structures, is effective only against enveloped viruses and has no effect on non-enveloped species. Furthermore, a pattern was discovered—the degree of antiviral effect depends on the origin and fractional composition of the HS [94].

In the work of K.D. Thiel et al. [95] conducted a comparative in vitro study of the activity of ammonium humate and fermentatively oxidized chlorogenic and caffeic acids against *human herpes virus types 1* and *2* (*HSV-1*, *HSV-2*). Ammonium humate (obtained from HA in bog water), as well as fermentatively oxidized diphenolic compounds, were shown to exhibit strong antiviral activity in vitro. Effective concentrations exceeded the cytotoxic range of these compounds.

The spectrum of activity of HA also includes the *H1N1* influenza virus, as discovered in the work of F.J. Lu et al. [96], where a study was conducted on the effect of synthetic humates (oxidative polymer of protocatechuic acid OP-PCA and HA) on the activity of the influenza virus. It was shown that these substances inhibit in vitro replication of the *A/WSN/33* (*H1N1*) influenza virus in Madin-Darby canine kidney (MDCK) cells at concentrations that do not cause cytotoxicity. OP-PCA inhibits virus-induced hemagglutination and fusion of the virus envelope with host cells, and HA inhibits the endonuclease activity of viral RNA polymerase. The authors [96] believe that HA can play the role of a chelating agent and inhibit viral RNA polymerase by binding to metal ions [96], i.e., in addition to the classical mechanism of action characteristic of all polyanionic compounds (binding to the surface structural proteins of the virion), after entering the nucleus of the affected cell, HA chelate Mn(II) ions, which are part of the viral RNA polymerase, which disrupts the cleavage of host RNA molecules by the viral endonuclease.

It is also known that HS exhibit high therapeutic efficacy in the treatment of hepatoma caused by the *hepatitis B virus* (*HBV*) [97]. It has been shown that HSs are capable of disrupting the main mechanism that *HBV* uses to survive in host cells—the formation of autophagosomes. Confirmation of the inhibition of the autophagy process in Hep G2.2.1.5 cells was provided by the results of Western blotting, which showed that the addition of HS to the cells led to a decrease in the levels of beclin-1, SIRT-1 and c-myc and an increase in the expression of caspase 3 and β-catenin [97].

Another study found that HS can act as potent antiprionogenic agents in the treatment of neurodegenerative disorders and exhibit a synergistic cytotoxic effect with β-amyloid protein in the SK-N-MC model of human nerve cells [98].

It is noted in the literature that the OH/OOH groups in the HA structure are also responsible for antiviral activity, since when deprotonated, HA is able to bind to the cationic sites of the virus, thereby inhibiting the attachment of the virus to the cell surface and preventing its replication [99].

V. Cagno et al. [88] conducted a study to examine the antiviral activity of shilajit against a group of viruses, including *herpes simplex virus types 1* and *2* (*HSV-1*, *HSV-2*), *human cytomegalovirus* (*HCMV*), *human respiratory syncytial virus* (*RSV*), *human rotavirus* (*HRV*) and *vesicular stomatitis virus* (*VSV*). Shilajit demonstrated dose-dependent inhibitory activity against *HSV1*, *HSV2*, *HCMV*, and *RSV* in vitro, but no activity against *HRV* and *VSV*. Moreover, the antiviral effect was not due to cytotoxicity. Commercial HA (Sigma, St. Louis, MO, USA), which exhibited the same spectrum of activity, was used as a control. Analysis of the test substance addition time and virucidal activity revealed that the inhibitory effect was primarily dependent on shilajit’s ability to interact with viral particles rather than cellular components, thereby preventing viral attachment to the cell surface. Shilajit also limited ongoing infection in vitro, as a decrease in viral yield was observed after several replication cycles [88].

The COVID-19 pandemic, which has infected more than 6 million people worldwide, has shown how limited the pool of antiviral drugs is [100,101]. Considering the broad spectrum of antiviral action of HS, P. Hajdrik et al. [102] investigated the possible antiviral activity of the commercially available food supplement ZnSeC-Humicin (Humic2000 Ltd., Budapest, Hungary) against the *SARS-CoV-2 B1.1.7* (*Alpha variant*) virus. A real-time quantitative polymerase chain reaction (RT-qPCR)-based viral replication inhibition test was used using different dilutions of the test substance in an in vitro infection model in Vero E6 cell culture. The results showed that the combination of HS with ascorbic acid, Se and Zn ions exerts an antiviral effect at a very low concentration range of the putative active ingredients. Even picomolar concentration ranges of HS, vitamin C, and Zn/Se ions in this composition were sufficient to achieve 50% inhibition of *SARS-CoV-2* virus replication in the applied virus inhibition test. The authors suggest that the antiviral effect may be due to the synergistic action of the components of this biologically active supplement on the cellular-viral system, which is consistent with literature data on the anticoronavirus activity of Zn, Se ions and natural HS [103,104,105].

The antiviral activity of FA has also been described in the literature [106,107,108,109,110], which states that FA effectively and safely destroy HIV and AIDS without damaging blood cells, and are effective against colds and flu, including respiratory viruses, retroviruses, influenza viruses, and herpes simplex viruses.

The ability of FA to influence viral replication and binding to host cells is described in the patent by J. Dekker et al. [79]. This study demonstrated the prevention of binding of six experimental viral cultures (*HSV*-1, *Human adenovirus type 2*, *Simian rotavirus SA 11*, *Poliovirus type 1*, *Coxsackie virus group A type 1*, *Coxsackie virus group B type 9*) at FA doses of 1.87 and 3.75 mg/mL. Viral replication was suppressed at concentrations ranging from 0.468 to 3.75 mg/mL. Limited inhibition of viral replication was observed at a concentration of 0.103 mg/mL in the case of *Simian Rotavirus SA 11*.

Thus, various antiviral properties noted for HS are linked to the activation of specific immune functions, primarily phagocytic immunity. Furthermore, the antiviral properties of HS are highly dependent on the specific chemical parameters of their structure.

### 2.4. Antitumor Action (Preclinical Studies In Vitro and In Vivo)

The antitumor properties of HA are associated with their electron-acceptor properties, due to which they are capable of producing ROS and causing apoptosis of cancer cells, as well as with the effect of HA on the cellular immune response, through which antitumor immunity is realized [111]. In the study by H. Kodama and Denso [111] humus extract inhibited the growth of transplanted L1210 tumors in DBA/2 mice. Animals given HS per os, experienced a delay in tumor formation and a decrease in tumor nodule weight. The antitumor effect was not due to a direct effect on tumor cells or the induction of apoptosis, as in vitro tests showed no significant inhibition of cell growth by the humus extract. No signs of apoptosis were also observed in L1210 cells cultured in the presence of humus extract.

A combination of lignin-derived HA with glucan administered intraperitoneally to mice showed significant (92%) inhibition of Ptas64 mouse breast cancer growth, as reflected by a reduction in tumor nodule weight, compared to glucan alone [12].

High cytotoxic activity of humus-derived HA was observed against human breast adenocarcinoma cells MCF7 in vitro [112]. The authors associate HA-induced apoptosis with a decrease in the expression of genes encoding the intracellular factor Bcl-2, which is a negative regulator of apoptosis, and an increased expression of caspase-3 [112].

A similar effect of HA on chronic myeloid leukemia K562 cells was observed by P. Mega Tiber et al. [113]. HAs have been shown to inhibit cell proliferation by decreasing the ratio of apoptosis regulatory proteins Bcl-2/Bax and increasing the levels of caspase-3 and caspase-9.

Humic acids can enhance the antiproliferative effects of known chemotherapeutic agents, allowing for a reduction in their effective dose. For example, HA produced by Sigma-Aldrich (USA) enhanced the antiproliferative effects of arsenic trioxide (As_2_O_3_) on HeLa and SiHa cervical adenocarcinoma cell lines. Growth inhibition occurred due to ROS-mediated cell damage and activation of apoptosis; an increase in the degree of DNA fragmentation and activation of caspase-3 were observed in the cells [114].

Fulvic acids have been shown to have effective antitumor activity, which is associated with their ability to inhibit cancer-causing viruses [87,109,115]. The study [116] evaluated the effect of FA on various cancer cells. Cells (Hep3B, HT29, and PC3) were treated with different concentrations of FA for 48 and 72 h, and proliferation was assessed using the MTT assay. It was shown that FA inhibit the proliferation of all cell lines used. It was also observed that Hep3B cells exhibited the highest sensitivity to 48 h treatment with an IC_50_ = 1.58–2.43 µg/µL. Notably, FA treatment also upregulated apoptotic genes at the mRNA level compared to the untreated control group.

A similar effect was demonstrated in the work by R.G.P.T. Jayasooriya et al. [42], using the example of Hep3B, LNCaP, and HL60 cancer cell death, FA also induced apoptosis in MCA-102 fibrosarcoma cells.

Another study by K. Pant et al. [117] demonstrates the antitumor and antiproliferative properties of FA. Huh7 cells were treated with various concentrations of FA (10–1000 µg/mL) for 24 h. Results were assessed using MTT and TUNEL assays. A dose-dependent inhibition of cell proliferation and NO production was observed, along with enhanced apoptosis and increased DNA damage.

Thus, HSs are promising natural substances capable of polarizing antitumor immunity and enhancing the antiproliferative effects of chemotherapeutic agents, which can be used to reduce the cytotoxic burden on cancer patients. Similar to the previous types of activity, a dependence on the specific structure of the HS has been observed.

### 2.5. Antioxidant and Antiradical Activity (Preclinical Studies In Vitro and In Vivo)

Systemic dysfunction of the entire organism is in one way or another associated with alterations in the functioning of an individual organ or organ system at the cellular level. Hypoxia is among the most common damaging agents that disrupt cellular homeostasis. Inadequate oxygenation ultimately leads to increased formation of reactive oxygen species (ROS), which damage critical cellular compartments. Consequently, interventions aimed at preventing disease development or combating existing pathologies should focus on disrupting ROS generation or neutralizing them. The high antioxidant (AO) activity of HS is their fundamental biological property, which determines such pharmacological effects as hepatoprotective, cardioprotective, nephroprotective, neuroprotective, cytoprotective, antitumor, antihypoxic, antitoxic, antidiabetic and others. Humic substances can act as proton donors, owing to the presence of phenolic hydroxyls and quinoidal fragments in their structure, and as free radical scavengers due to their high paramagnetism [1,118,119,120]. The implementation of AO action through multiple mechanisms simultaneously, along with the capacity to neutralize free radicals in both enzymatic and non-enzymatic lipid peroxidation processes (a property not characteristic of all antioxidants), underscores the therapeutic advantage of using HS [121,122].

*Activity of HS relative to various reactive oxygen species and antioxidant enzymes (preclinical studies in vitro)*

In reference [123] HA are described as molecules with low AO activity (approximately 20%) towards the superoxide anion radical (O_2_^−•^) and high AO activity (approximately 50%) towards the hydroxyl radical (HO•). The authors in [123] report a decrease in superoxide dismutase (SOD) activity and a reduction in glutathione content upon mixing HA with mitochondria (in models of enzymatic and non-enzymatic AO systems of liver mitochondria and in vitro). However, they noted that the activity of glutathione peroxidase and glutathione reductase remained unchanged. The ability of HA to enhance the activity of AO enzymes, attributed to their electron-acceptor properties towards free radicals, is also described in the study of S.A. Visser [124].

As a result of in vitro experiments (the ABTS assay and the 3T3-L1 fibroblast cell line assay using the fluorescent probe 2,7-dichlorodihydrofluorescein diacetate (DCFDA)), it was discovered that various HS (HA from peat and coal, FA) did not exhibit a pro-oxidant effect and possessed high AO activity [125].

The AO properties of FA also allow for their recommendation for use in the pharmaceutical and food industries as an accessible source of natural antioxidants [126,127,128]. In the work of N.C. Rodríguez et al. [128] their antiradical and AO activity was evaluated against O_2_^−•^, hypochlorous acid (HOCl), hydrogen peroxide (H_2_O_2_), HO•, peroxynitrite (ONOO^−^), and singlet oxygen (^1^O_2_). It was shown that FA exhibits an activating effect, but less than nordihydroguaiaretic acid (NDGA), ascorbic acid, pyruvate, dimethylthiourea (DMTU), penicillamine, and glutathione (GSH). In the study by Y. Gao et al. [129] investigating the AO properties of FA, an increase in the expression of reduced glutathione, catalase, and SOD, and a decrease in lipid peroxidation (LPO) markers were observed. Similar results are shown in the work by Shikalgar et al. [130]. It is also noted that the high AO potential of FA to inhibit O_2_^−•^ and other ROS is associated with their ability to prevent uncoupling of electron transport in liver mitochondria [123].

In the study [2], during the investigation of the AO activity of coal HS, the ability of HS to reduce levels of stable free radicals DPPH and ABTS^•+^ was demonstrated, comparable to the reference compounds Dihydroquercetin and Trolox (synthetic water-soluble analog of vitamin E). The investigated HS sample also exhibited a high capacity to inhibit free radicals O_2_^−•^ and HO• in model systems, comparable to the reference compounds Ascorbic acid and Mannitol. This is a crucial property, as O_2_^−•^ and HO• are the most dangerous free radicals, causing oxidative damage to DNA, proteins, and membrane lipids. They can bypass endogenous AO defense systems by inactivating certain specific enzymes that protect the body from oxidative damage, particularly glutathione peroxidase, catalase and others [131,132,133]. Furthermore, under the action of O_2_^−•^, Fe^3+^ ions bound to ferritin within cells can be released as Fe^2+^ ions, which can generate HO• via the Haber-Weiss reaction [134]. Additionally, in this study [2] the AO properties of HS were investigated using cathodic voltammetry and it was noted that the catalytic activity value of coal HS (0.91 µmol/L·min) is comparable to that of Ascorbic acid (1.15 µmol/L·min) and exceeds that of Dihydroquercetin (0.78 µmol/L·min). A potential mechanism for the AO activity may be related to the ability of quinoid groups to participate in the electrochemical reduction in O_2_ (EO_2_ process). High chelating properties of HS were also established with a ferrozin-Fe^2+^ complex, which is highly significant as the ability to chelate variable-valence metals is one of the most crucial mechanisms of AO activity for biologically active substances. The authors [2] concluded that the AO and chelating activity of HS are due to the high content of phenolic and quinoid groups, as well as semiquinone-type radicals, within their structure.

There are also studies where, through chemical modification involving the introduction of hydroquinoid and hydronaphthoquinoid centers into the structure of HS (via oxidative copolymerization of phenols), new substances with high AO activity can be created for various purposes [135]. The authors demonstrated in their work that the original HA and their naphthoquinoid derivatives possessed high acceptor capacity, while FA and their hydroquinoid derivatives exhibited both high donor and high AO capacity.

*Antioxidant activity in the manifestation of protective properties of humic substances (preclinical studies in vitro and in vivo)*

The stimulation of free radical processes is a non-specific factor that provokes cell death in any organ and underlies the pathogenesis of many socially significant diseases (stroke, myocardial infarction, cancer, etc.). Humic substances, administered after focal cerebral ischemia, due to their pronounced AO properties, reduced neuronal necrosis. Evidence for this included an increase in SOD and NRF1 levels and a decrease in malondialdehyde (MDA) concentration, as well as reduced cerebral edema, vacuolization, degeneration, and destruction of neural elements [136].

A study [137] demonstrated that HS administration preceding acute ischemic kidney injury reduced biochemical parameters such as total AO status (TAS), total oxidative status (TOS), and ischemic modified albumin (IMA), but increased the oxidative stress index (TOS/TAS). Thus, the obtained data indicated a reduction in renal reperfusion injury by preventing the development of oxidative stress. It is also noted that HS administered during the therapeutic window for focal cerebral ischemia (hypoxic–ischemic encephalopathy), due to their AO properties, reduce the degree of excitotoxicity and the intensity of lipid peroxidation. Evidence of these favorable changes is the decreased immunoreactivity of caspase-3 in neuronal cytosol [138].

In another study, the Polish preparation Tolpa Peat Preparation, which is a complex of HA from raised bog peat, suppressed LPO in mitochondria isolated from human placenta, as evidenced by a decrease in the level of MDA, which are formed during the degradation of polyunsaturated acyls within the organelle’s membrane lipids [139]. The efficacy of HS in this regard was comparable to that of vitamin E, a comparator drug [139]. The ability of HS, due to their AO properties, to protect the main components of the karyoplasm, particularly DNA, from ROS (superoxide anion radical and hydroxyl radical are particularly hazardous to these macromolecules due to the oxidation of nitrogenous bases to oxo-derivatives) forms the basis of their application as a primary agent, as well as in combination with other chemotherapeutic drugs, for the treatment of malignant neoplasms [140]. However, there is evidence that the use of HS during chemotherapy and radiotherapy can reduce the severity of side effects, as these natural molecules reduce damage to the genetic material in intact cells [141]. But there is also information suggesting that the anticancer properties of HS may not only be related to their AO effects but also to their ability to induce the production of ROS and NO by cells. For instance, study [141] showed that incubation of Huh-7 cells with HS at various concentrations led to a dose-dependent increase in their production of ROS and NO, which consequently activated apoptosis and inhibited proliferation processes. Similar results regarding the antitumor effects of himatomelanic acids have been presented in the work by I. Jurcsik et al. [142].

In the study [2], an investigation into the AO properties of coal HS in HepG2 cell cultures was conducted, assessing the ability of HS to reduce the fluorescence intensity of the fluorescence probe DCFDA after stimulation of free-radical processes by adding oxidizers (hydrogen peroxide, tert-butyl hydroperoxide, Fe^2+^ ions). It was determined that pre-incubation of HepG2 cells with the HS sample (25 µg/mL) reduced intracellular ROS levels following oxidative stress induction by all types of oxidizers. This indicates a high cytoprotective potential of HS for stimulating/enhancing cellular AO defense systems.

High AO activity of HS was demonstrated in the laminectomy followed by traumatic spinal cord injury (TSCI) test. The administration of HS after TSCI procedures showed a significant reduction in the severity of edema, the content of polymorphonuclear and mononuclear leukocytes, as well as a significant (*p* < 0.05) decrease in the level of paraparesis [143]. Humic acids administered intraperitoneally to Wistar rats after surgery modeling traumatic spinal cord injury reduced the overall serum oxidant status 24 h post-intervention. Furthermore, histopathological assessment of the spinal cord at the injury site revealed a significant reduction in edema (*p* < 0.001), hemorrhages, polymorphonuclear leukocytes, mononuclear leukocytes, and macrophages compared to the control group, along with a substantial restoration of paraplegia levels in the experimental groups [143].

*Antioxidant activity in the manifestation of antidiabetic properties of humic substances (preclinical studies in vitro and in vivo)*

A study was conducted to investigate the effect of FA on streptozotocin-induced type 2 diabetes mellitus [144], where it was found that FA can reduce hyperglycemia and increase SOD activity. Additionally, there are studies [145,146] in which the authors established the mechanisms of hypoglycemic activity and antidiabetic effects of HS in models of alloxan-induced and streptozocin-induced diabetes. Presumably, the mechanism for realizing the antidiabetic effect in alloxan-induced diabetes is the high immunomodulatory activity of HS (they prevent the infiltration of activated macrophages and lymphocytes into the inflammatory site, which are sources of cytotoxic oxygen radicals), and in streptozocin-induced diabetes, it is the ability to increase SOD activity in the β-islet cells of the pancreas. The authors [144] drew the conclusion about the ability of FA to prevent free radical damage to pancreatic β-cells.

It was also mentioned above that the authors of the study [36] suggested the possibility of using humic water in the maintenance therapy of endothelial dysfunction in diabetes mellitus.

*Antioxidant activity in the manifestation of antitoxic properties of humic substances (preclinical studies in vitro and in vivo)*

Under conditions of oxidative stress induced by oral administration of hydrogen peroxide to Sprague-Dawley rats, the AO effect of HA derived from compost were evaluated by measuring the levels of AO enzymes SOD and glutathione peroxidase (GPx) in the animals’ serum and the marker of lipid peroxidation MDA in liver homogenate. A decrease in SOD, GPx, and MDA were observed, indicating protective effects of HA against induced oxidative damage [147].

The AO effect of HA was also demonstrated in rats with oxidative stress induced by aflatoxins. Oral administration of HA led to an increased AO status (decreased MDA levels and increased glutathione levels) in the tested organs (liver, kidneys, testes, and brain) [148].

In an in vivo experiment, HS showed the ability to neutralize the harmful effects of vomitoxin (one of the most commonly found mycotoxins in unprocessed grains) when administered at a dose approximately twice the permissible daily intake. The manifested antitoxic effect is associated with the high AO activity of HS, due to the activation of enzymes forming the endogenous AO system of the cell, primarily SOD and glutathione-S-transferase (GST) [21]. Moreover, it was found that HS reduced the bioavailability of the toxin by adsorbing it onto their surface due to their colloidal properties [21].

The AO properties of HS from weakly mineralized sulfide muds (peloids) were investigated using initiated oxidation of 1,4-dioxane as a model reaction [149]. The kinetic characteristics of HS oxidation were determined in terms of effective inhibition rate constants of oxidation. It was established that all HS possess AO activity, with the highest indicators recorded for hymatomelanic acids.

*Study of antioxidant and antiradical mechanisms of humic substances (studies in vitro)*

In addition to studies primarily aimed at proving the efficacy of HS in the therapy of specific diseases, there are several works dedicated to the investigation of HS pharmacodynamic parameters. For instance, in a series of experiments to determine the AO activity of HS [123], a fundamentally new possible mechanism for realizing the AO effect was proven: the HS molecule contains quinoid-type fragments (similar in structure to coenzyme Q, a non-protein component of the mitochondrial electron transport chain). Due to structural similarity, accepted electrons are transported within the HS molecules without the participation of mitochondrial enzymes, which ultimately leads to impaired reduction in formed peroxides [123].

The main objective of the work by O.I. Klein et al. [150] was to evaluate the AO activity of a large number of HS (25 samples of HS from soils, peat, coal and water HS, as well as humic-like substances) with significantly different structures was assessed using the Oxygen Radical Absorbance Capacity (ORAC) assay, elemental analysis, ^13^C-NMR spectroscopy, and the Folin–Ciocalteu method for quantifying total phenolic content (compared to ascorbic acid and vitamin E). The results were processed using stepwise multiple linear regression (atomic C/N ratio, phenolic content, O-substituted methine and methoxy groups). The obtained results demonstrated that the AO activity of HS depends on both phenolic and non-phenolic fragments in their structure, including carbohydrate fragments.

**Figure 5:**
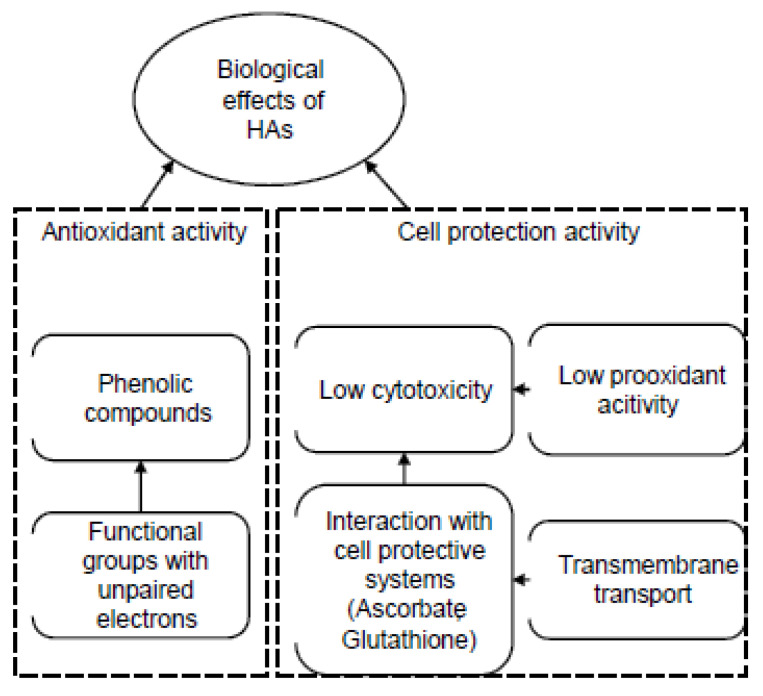
A hypothetical ontology-based model of biological activity of humic aids [151].

A deep fundamental study of the AO properties of HA isolated from different types of peat, in relation to their physicochemical structural parameters, was also conducted [151], presented in Figure 5.

In this study [151], the structural parameters of HA were investigated using electron, fluorescence, infrared, and ^13^C-NMR spectroscopy, titrimetric analysis, C, H, N, O elemental analysis, and gel chromatography. Antioxidant and antiradical activities were studied using physicochemical analytical methods: electron paramagnetic resonance, cathodic voltammetry, spectrophotometric tests with ABTS^•+^ and DPPH radicals, determination of superoxide anion radical and hydroxyl radical activity, as well as investigation of chelating ability. Intracellular ROS production was assessed using the fluorescent probe DCFDA, intracellular ROS production was induced using two common pro-oxidants (tert-butyl hydroperoxide and Fe^2+^ ions). Cytoprotective activity was investigated using a neutral red-based cytotoxicity assay in a 3T3-L1 cell culture across a wide range of concentrations. The authors [151] proposed an ontological model (Figure 5) of AO and cytoprotective activity of HA based on experimental data and numerical models, which opens the way for further research into the biological effects of HA and provides a useful tool for numerical modeling of these effects. The high AO and cell-protective activity of HA make them a promising natural source for new pharmaceutical substances.

**Figure 6:**
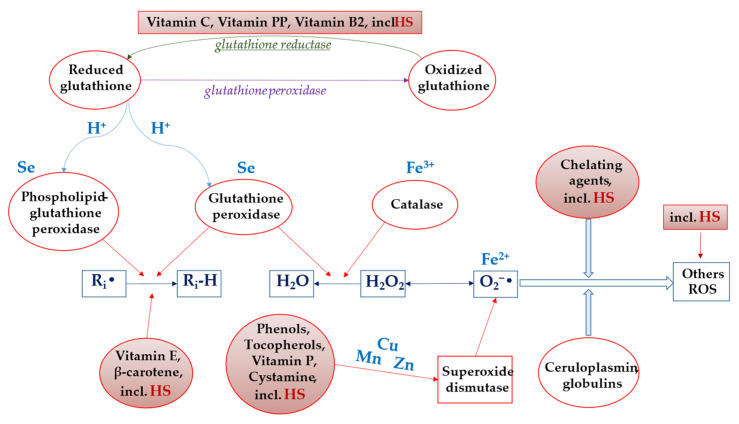
A hypothetical scheme of antioxidant activity of humic substances.

Thus, based on numerous reports in the literature sources and our own experimental data from many years of HS research, it is possible to propose a hypothetical scheme of the AO activity of HS, shown in Figure 6.

### 2.6. Cardioprotective Action (Preclinical Studies In Vivo)

The beneficial effects of HS on the myocardium are described in the works of T.V. Lasukova et al. [152,153]. For instance, in an isolated perfused Langendorff heart model under normoxic conditions, direct dose-dependent cardiovascular effects of HS were observed. Upon addition of HS to the perfusate, coronary blood flow normalized, and end-diastolic pressure decreased. The cardioprotective properties of HS were also evident in the heart muscle under conditions of global ischemia–reperfusion: prophylactic administration of HS prior to ischemia reduced reperfusion contracture, contributed to a decrease in necrotic cardiomyocyte death, and promoted a more effective recovery of contractility. The mechanism of HS action on the myocardium, detached from the regulatory systems of the whole organism, is attributed by the authors to the activation of NO-synthase by HS. It was demonstrated that upon the addition of the selective NO-synthase modulator L-NAME to the perfusion solution, the production of the vasoactive component NO was observed [152,153].

The application of HS in cardiology may be linked not only to their favorable influence on neovascularization processes and their vasodilatory effect but also to their ability to improve several important hematological parameters. Thus, studies [26,154] have demonstrated that HS shorten prothrombin time (inhibiting the prothrombin complex—active factors II, VII, and X), reduce glucose, total cholesterol, and lipid levels, but increase hematocrit and HDL cholesterol levels.

A study [65] demonstrated that in animals administered the TPP following coronary artery occlusion, which resulted in myocardial infarction, restoration of blood flow in the ischemic zone occurred due to HS-induced TNF-α. Thus, through its pro-angiogenic and cardioprotective actions, TPP from HS mitigated the development of cardiomyopathy [65]. Owing to this valuable therapeutic effect, TPP has found application in cardiology for geriatric patients with ischemic heart disease [66]. Moreover, TPP increases the viability of mononuclear leukocytes and induces neovascularization processes in patients with this diagnosis. The effect of HS on angiogenesis also plays a key role in other physiological processes, including embryogenesis and wound healing [64]. In studies by C.E. van Rensburg et al. investigating the anti-inflammatory properties of HS, it was also established that HS possess cardioprotective and pro-angiogenic effects [19].

The cardioprotective properties of FA are described in the study of T.S. Shikalgar [130] using a model of isoprenaline cardiotoxicity It was found that the daily use of FA at a dose of 300 mg/kg for a month contributed to the prevention of such cardiotoxic effects as electrocardiogram and hemodynamic disturbances, expression AO and cardiac markers.

Thus, it can be assumed that the implementation of cardioprotective effects is carried out due to the influence of HS on the immune and antioxidant systems of the body.

### 2.7. Hepatoprotective Action (Preclinical Studies In Vivo)

Studies on the therapeutic effects of HS on hepatocytes are of significant importance, stemming from both the crucial functional role of the liver and the potent protective activity of HS against hepatic cells when exposed to hepatotropic toxins of various chemical natures, including pharmaceuticals. The high hepatoprotective potential of HS is attributed to their AO and antitoxic properties, characterizing them as inducers of the microsomal system and activators of plastic metabolism [76].

In models of acute intoxication induced by psychotropic drugs Hexobarbital and Medinal, at doses approaching LD_50_, a reduction in the duration of hypnosis was observed. This is due to the ability of HS to induce the activity of cytochrome P450-dependent monooxygenases, primarily from the CYP1IA subfamily [155]. Humic substances can protect the hepatic parenchyma from the effects of toxic agents not only directly, by modulating the redox status or enhancing the structural-metabolic parameters of its constituent structural units, but also indirectly, by adsorbing toxins from the intestinal lumen. For instance, in a study [156], HS mitigated the oxidative stress and inflammatory responses induced by aflatoxin B1. This was achieved through their interaction with the toxin, forming macromolecular complexes incapable of penetrating the epithelial cell membrane. Furthermore, the authors attribute the hepatoprotective effect to the modulation of intestinal microbiota by HS, specifically the restoration of the Firmicutes/Bacteroidetes ratio [156].

In another experiment [157], HS prevented the cytolytic action of carbon tetrachloride (CCl_4_) on liver cells. This effect was due to their potent AO properties, which neutralized the metabolic products (free radicals) of this hepatotropic toxin. Consequently, this action led to a reduced intensity of lipid peroxidation, near-complete cessation of phospholipid layer destruction in hepatocytes, diminished severity of the cytolytic syndrome, and improved hepatic excretory function, thereby preventing the development of fibrotic structures [157].

The ability of HS to protect cellular mitochondria, the primary energy organelles, from uncoupling of oxidative phosphorylation has been demonstrated in a murine model of normobaric hypoxia with tissue hypercapnia in the liver and brain of mice [158,159]. The antihypoxic activity of HS was comparable to that of the reference drug Dihydroquercetin in neurons and exceeded it in hepatocytes. The observed normalization of oxidative phosphorylation under the influence of HS is linked to the restoration of respiratory chain component activity (succinate and NAD-dependent dehydrogenases) [158,159].

In another study, HS mitigated the effects of dystrophy induced by the administration of Sovol (a mixture of tetra- and pentasubstituted diphenyls), also by restoring the cellular redox homeostasis [160]. In a study [161], oral administration of HS during iron-induced hepato- and cardiotoxicity resulted in reduced organ damage, attributed to both the direct binding of Fe(II) ions by HS and the enhanced activity of endogenous cellular AO system components, particularly the enzyme SOD. It has also been demonstrated that the AO and antiradical activity of HS is exhibited towards the most reactive and toxic forms of oxygen—the superoxide anion radical and the hydroxyl radical, which are neutralized through reduction mediated by the phenolic and quinoidal fragments within the HS structure [1,149].

In an experiment involving lipopolysaccharide (LPS)-induced inflammation, hepatoprotection observed with HS administration was accompanied by a decrease in intracellular enzyme levels, primarily alanine aminotransferase (ALT), and the resolution of hyperglycemia. This was a consequence of HS influencing the production of pro-inflammatory cytokines, specifically IL-2 [13].

Prophylactic administration of peat HA, β-glucan, and their combination prior to xenobiotic-induced hepatotoxicity (induced by ethanol, CCl_4_, and lipopolysaccharide from *E. coli*) demonstrated a reduction in markers of necrotic liver injury, including aspartate aminotransferase (AST), ALT, and alkaline phosphatase in the serum of BALB/c mice, as well as MDA and SOD levels in the liver. At the same time, the combined use of glucan-HS exhibited a synergistic cytoprotective effect, which the authors attributed to a more efficient restoration of glutathione levels and the combined AO activity of each individual component [162].

In a study of the drug TPP administered following partial liver resection, laboratory animals showed an increase in liver mass, resulting from enhanced activity of ornithine decarboxylase, a key enzyme in polyamine synthesis and a stabilizer of nucleic acid molecules [163].

### 2.8. Regenerative Action (Preclinical Studies In Vitro and In Vivo, Clinical Trials)

Traumatism has become a significant medico-social problem, especially following the extensive automation of most spheres of life. Damage to organs and tissues is one of the “top three” causes of mortality among the economically active population. Moreover, traumatism due to high cost of treatment, the redistribution of significant financial flows to the social sphere, causes great damage to the economic development of any country. Therefore, governmental and affiliated medical institutions dedicate considerable attention to organization and implementation of measures to prevent injuries and the development of immediate and delayed complications after them. Therefore, the search for substances that positively influence the key stages of the reparative process and are more accessible compared to instrumental methods remains a pressing task.

Humic substances are widely used in the treatment of various skin and mucous membranes diseases, and to accelerate wound healing both externally and internally. The regenerative and wound-healing effects of HS are based on their ability to form hydrogen and covalent bonds with biopolymers such as collagen [22].

*Wound healing properties* *of applied externally of humic substances*

In the work by Y. Ji et al. [164] sodium humate was investigated as a treatment for skin wound healing in Sprague-Dawley rats. Sodium humate demonstrated wound-healing ability in rats by accelerating wound reduction and increasing hydroxyproline content in the tissues. The authors [164] identified that the wound-healing effects of sodium humate may be mediated by the TGF-β/Smad signaling pathway. The transforming growth factor beta (TGF-β) signaling pathway is closely related to wound healing and scar formation, with Smad proteins functioning as intracellular signal mediators for the TGF-β superfamily.

A biomaterial based on HA from Sigma (USA) and alginate suppressed in vitro the expression of type I collagen, which plays a role in scar formation, and stimulated the expression of type III collagen, which is involved in wound healing [37]. This effect was observed in the L929 mouse fibroblast cell line without affecting cell viability. Thus, an HA-based hydrogel seems to be a promising material for scarless wound healing [37].

Topical applications of HS to rat skin enhance proliferation processes and active water, protein, and lipid metabolism. The skin exhibits an increased number of fibroblasts and histamine, leading to accelerated healing processes [165]. A wound-healing effect of HS, monolayer regeneration, and rapid wound healing were identified in a scratch model [13]. The mechanism of wound healing is linked to the paracrine stimulation of cytokines rather than autocrine stimulation of proliferation, as indicated by the absence of increased HaCaT cell proliferation [13].

It has been demonstrated that HA derived from peat can improve oral wound healing [166]. The application of HA to palatal wounds in Wistar rats was observed to promote more intensive healing than both the control (saline solution) and chlorhexidine gluconate.

The protective effect of HS against UV-induced cellular damage [167], is of significant interest, their protective effect against UV-induced cytotoxicity is comparable to Solcoseryl and Beloderm. An important practical property of HS—protecting the organism from radiation exposure—was discovered in a study [168]. For instance, HS added to lymphocytes of thyroid cancer patients immediately after γ-irradiation reduced the number of aberrant cells. The authors [168] associate this effect with the activation of DNA repair and repopulation processes by HS. Study [167] provides data indicating that a composition of HS and castor oil, applied 24 h post-UV-B irradiation to promonocytic model cells (U937 cell line), increased their survival due to the ability of each component to absorb light in the visible spectral range. Thus, HS can be considered as promising components for the creation of cosmetic products that slow down photochemical aging of the skin [167].

*Biostimulating effect* *of humic substances*

Humic substances are capable of increasing the tensile strength of rat heel tendons, enhancing the mechano-chemical resistance of collagen fibers, and accelerating their maturation processes [169]. The biostimulatory effect of HS has been demonstrated in rats with laparotomy, showing a significant reduction in adhesion formation [170]. Clinical results show that HSs are able to stimulate osteoclastic resorption of transplanted bones and hydroxyapatite used for bone reconstruction [171].

Humic preparations derived from peat are effective in treating rheumatoid arthritis, eczema, and osteoarthritis (complicated by cartilage destruction) [172,173]. A stimulating response in smooth muscle contractile activity has been identified through dopaminergic mechanisms (involving D2-opioid receptors that induce itching) and α2-adrenergic stimulation [174]. Therefore, HS preparations, acting as neurogenically mediated mediators of hyperemia and inflammation, are of considerable interest for the therapy of rosacea through the inhibition of serotonin reuptake and stimulation of α-adrenoreceptors [175].

*Wound healing properties of fulvic acids*

Fulvic acids are also able to exert a regenerative effect. In some studies, accelerated healing of ulcerative defects and wounds has been observed due to enhanced fibroblast proliferation and activation of tissue hyaluronidase [176]. The potential of FA as a wound healing and anti-inflammatory agent is described [60,177], in these studies, FA applications were used topically for the treatment of hematoma, phlebitis, desmorrhea, myogelosis, arthrosis, polyarthritis, osteoarthritis, and osteochondrosis. It is noted that FA ions actively influence the capacity of dermal cells for healthy growth, regeneration, and division [178], thereby promoting improved skin condition and elasticity. Furthermore, FA’s influence on bone tissue restoration impedes the development of osteoporosis and contributes to skeletal strengthening, thereby reducing the rehabilitation period following surgical interventions and prolonged chronic diseases [178].

In the study of R. Sabi et al. [54] topical FA application to a wound infected with *Staphylococcus aureus* was found to inhibit the synthesis of interleukins and prostaglandins. A reduction in wound size and decreased infection progression were observed. Similar results are described in the article by Y. Zhao et al. [179] which evaluated the efficacy of wound healing in infections caused by *Methicillin-resistant Staphylococcus aureus* and *Pseudomonas aeruginosa*. The results showed that on the 3rd day post-infection, the upregulation of the pro-inflammatory cytokine interleukin-6 (IL-6) was significantly attenuated (for comparison, in an untreated wound the increase reached 2485 times, and in a treated wound—791), and accelerated wound healing was observed on days 6 and 10.

The scientific study by C.E.J. Van Rensburg et al. [78] demonstrated that oxifulvic acid (in the form of a 5.3% cream) had a positive effect in the topical treatment of pyotraumatic dermatitis in animals and also inhibited contact hypersensitivity in mice.

In the article of J.J. Gandy et al. [180] clinical trials on the use of FA in the treatment of eczema of varying severity in 36 patients aged 2 years and older are described (applications twice daily with a 3.5% FA solution, pH 4.8). The highest efficacy in eczema treatment was established for symptoms of overall severity and erythema. No side effects on the hematopoietic or hepatic systems were identified, only transient local burning.

In the article of Y. Shenyuan [181] the efficacy of topical application (compresses) of FA for the treatment of autoimmune arthritis and systemic lupus erythematosus is described, noting its strong immunomodulatory, AO, and anti-inflammatory effects.

In the study by I.A. Schepetkin et al. [182] it is concluded that oral administration of moomiyo FA will be effective in the treatment of gastritis, diarrhea, stomach ulcers, dysentery, colitis and diabetes mellitus.

### 2.9. Detoxifying Action (Preclinical Studies In Vitro and In Vivo)

The presence of oxygen- and nitrogen-containing functional groups on the surface of HS molecules accounts for their high chemical reactivity and ability to engage in ion-exchange and complexation reactions. This means they can facilitate the binding of many toxic metals and xenobiotics and thus be transported with them through the body’s detoxification systems. Humic substances inactivate polyvalent metal ions (lead, cadmium, copper, zinc, aluminum, manganese, iron, etc.) [183,184,185,186,187,188,189,190], block the development of invasive processes, bind bacteria and viruses [26], aflatoxins [156,191], mutagens [192], pesticides, and ammonia [193], as well as mono- and polycyclic aromatic compounds [191,192,193], also prevent the adverse effects of ionizing radiation [194].

*Interaction of humic substances with inorganic toxicants*

It has been established that upon simultaneous administration of HS and inorganic lead, the resorption and accumulation of the toxicant in the liver decreased by an average of 30%, in the kidneys by 44%, in muscles by 58%, and in bone tissue by 51% [184]. The antitoxic properties of HS are also demonstrated against other heavy metals. For instance, in two independent studies [185,186] conducted on the same biological model, *Salmo trutta fario*, L., HS prevented the development of oxidative stress induced by the entry of CdCl_2_(II) and MnCl_2_(II) into the organism’s internal environment. This was evidenced by a reduction in the level of the primary marker of inflammation—MDA, as well as the normalization of GPx and SOD levels [185,186]. The ability of a complex preparation based on HS to effectively bind cadmium into a stable coordination compound during prolonged exposure to the organism (in metallurgical workers and smokers) is also described in [194], where the authors recommend using this preparation for the removal of heavy metals from biological media.

Several studies [189,190] have noted that FA, isolated from soils and aquatic environments, are highly reactive with metals and form strong complexes with Fe^3+^, Al^3+^, and Cu^2+^. In addition to these fundamental properties, HS can complex with hydrophobic compounds, making them soluble in water [195].

The application of HS as a component of efferent therapy offers several advantages over other enterosorbents, such as activated charcoal, due to its capacity to engage in not only physical interactions with the adsorbate via Van der Waals forces but also ion-exchange, ligand exchange, and redox reactions [26,76].

*Interaction of humic substances with organic toxicants*

The author collective A.V. Buzlama et al. [76] suggest that upon oral administration, HS can form a barrier layer on the gastrointestinal tract surface, thereby retaining water and preventing its loss through the intestines, thus providing mucosal protection against various aggressive factors. Through the summation of these favorable effects, HS exert several pharmacological actions: detoxification, enterosorption, gastroprotection, and antidiarrheal effects [76]. In a prophylactic application of HS in rats with experimentally induced gastric and duodenal ulcers by ethanol, a reduction in mucosal ulcerogenesis and accelerated healing processes were observed [67].

Humic substances have also been used as hemostatic agents in the treatment of gastric and duodenal ulcers [196], and for treating metabolic disorders of the digestive system [197], with noted absence of side effects and complete drug elimination. The anti-ulcerogenic effect of HS [76,198] is realized through their macrocolloidal properties and their ability to reduce the permeability of histochematic barriers, creating a protective barrier layer on the gastric mucosa. Humic substances are of great interest in this regard [76,198], particularly because substances with anti-inflammatory properties typically exhibit ulcerogenic effects, unlike HS, which possess both anti-inflammatory and anti-ulcerogenic actions. This means that HS possess the ability to autonomously mitigate the severity of NSAID-induced gastropathy and stress-induced ulcerogenesis [198]. A preparation based on Fe(II) ions chelated with HS exhibited greater bioavailability and was therefore recommended for use in veterinary medicine for the combined therapy of intestinal and iron-deficiency syndromes [199].

It has been established [192] that HS inhibit the mutagenicity of benzo[α]pyrene, 2-aminoanthracene, 2-nitrofluorene, and 1-nitropyrene in the *S. typhimurium* test. The desmutagenic effect was attributed to the adsorption of mutagens onto HS molecules [200].

Peat-derived HA have the ability to reduce animal mortality induced by the administration of various toxic substances (strychnine, phenylhydrazine, sodium nitroprusside, carbon tetrachloride) at lethal doses [159].

In a study of the detoxifying properties of HS (Lignohumat) [155] using a model of acute poisoning with the antipsychotic drug clozapine, reduced lethality, improved clinical condition of the animals, and decreased hepatocyte cytolysis were observed.

Upon intoxication with oxidized oleic acid and mesoxalylurea, it was established [155] an antiradical mechanism of HS’s detoxifying activity, attributed to their reducing properties.

*Interaction of humic substances with mycotoxins*

In an in vivo experiment, HS demonstrated the ability to mitigate the harmful effects of vomitoxin (one of the most frequently detected mycotoxins in unprocessed grain), administered at a dose approximately twice the permissible daily intake. The exhibited antitoxic effect is linked to the high AO activity of HS owing to the activation of enzymes that form the cell’s endogenous AO system, primarily SOD and glutathione-S-transferase [21]. Furthermore, it was discovered that HS reduced the bioavailability of the toxin by adsorbing it onto their surface due to their colloidal properties [21].

### 2.10. The Influence of Humic Substances on the Bioavailability of Drugs (Preclinical Studies In Vivo)

In addition to improving micronutrient absorption, HA and FA mediate drug delivery [201,202]. A study using a rat “everted sac” model with the anticonvulsant drug carbamazepine demonstrated that administration of a carbamazepine complex: FA (1:2) increased absorption, along with the concentration of carbamazepine in plasma [201]. In yet another study by a team of authors, M.A. Mirza et al. [202], the potential of mumie-derived HA for oral delivery of carbamazepine is described. For ex vivo studies, rat intestinal permeability was assessed, and a pharmacodynamic assessment (using maximal electroshock method) was conducted. Molecular modeling and instrumental analysis confirmed the localization of carbamazepine within the HA complexing agent. Enhanced solubility (∼1742%), delayed release (∼78%), improved permeability (∼3.5-fold), and intensified pharmacodynamic responses were also observed. The authors [202] concluded that HA can be utilized as complexing agents for antiepileptic drugs and other classes of poorly water-soluble medications.

### 2.11. The Influence of Humic Substances on the Microbiome (Clinical and Preclinical Studies)

Scientists from Charité Universitätsmedizin, Berlin [203] conducted a study on the influence of HA in the medicinal product Activomin^®^ (WH Pharmawerk, Weinböhla, Germany) upon oral administration, on the concentration and composition of the colonic microbiome. The results obtained indicated an increase in the total concentration of intestinal microbiota from 20% on day 10 to 30% on day 31, remaining stable until day 45 (32%). This increase in concentration for each individual was due to the growth of previously existing groups, while the individual microbial profiles of the patients remained unchanged. Similarly, bacterial diversity remained stable. The concentrations of 24 out of 35 microbiomic groups increased from 20% to 96%, including increases in the concentrations of pioneer groups such as Bifidobacteriaceae, Enterobacteriaceae, and *Clostridium difficile*. The authors concluded that HA exert a profound impact on the health of the colonic microbiome and may represent potentially interesting substances for the development of drugs that control the innate intestinal microbiome [203].

A positive influence of HS on the gut microbiome was also noted in [156]. In this study, it was observed that HS reduced aflatoxin B1-induced oxidative stress and inflammatory reactions, as to the formation of macromolecular complexes between HS and the toxin, which were unable to penetrate the enterocyte membrane. The authors [156] attribute the hepatoprotective effect to the modulation of gut microbiota by HS, specifically the restoration of the Firmicutes/Bacteroidetes ratio.

### 2.12. Adaptogenic Effect (Preclinical Studies In Vivo)

Over the past century, there has been a fundamental transformation in the structure of morbidity and mortality. Infectious diseases, with the exception of a small number of viral pathologies, have ceded their leading position to equally dangerous nosologies such as ischemic heart disease, hypertension, oncological diseases, gastric and duodenal ulcers, iron-deficiency anemia, diabetes mellitus, mental disorders, and others. Both the etiology and pathogenesis of these diseases share common features, where the decisive role in their onset and development belongs to an intense and prolonged stress response, provoked by external extreme environmental factors. This implies that enhancing the resistance of a healthy organism to counteract the detrimental effects of stress factors is one of the principles for preventing major non-infectious diseases. To mobilize the body’s reserve forces, adaptogenic and psychostimulant drugs derived from plant materials are often used. It is known [204] that the use of HS (as part of mumie) as adaptogens began over 3000 years ago and continues to this day, with many adherents even among proponents of high-tech medicine.

The literature describes that the combination of AO, immunotropic, detoxicating, antibacterial, and antiviral effects of HS underpins their ability to act as stress correctors and adaptogens, enhancing the nonspecific resistance of animal and human organisms [76]. A.V. Buzlama et al. [76] described the mechanism of adaptogenic effects: HS contribute to the activation of phagocytic immunity, and they can also serve as phenolic antioxidants and detoxificants. Simultaneously, they can exhibit pro-oxidant activity; however, low-level pro-oxidant activity initiates the body’s AO defense system and stimulates phagocyte activity. This process is complemented by other aspects of immuno-oxidant mechanisms of adaptogenic stress-correcting action. Low-molecular-weight fractions of HS readily cross histohmatic barriers and enter the bloodstream, being transported by it to the liver and other organs. Higher-molecular-weight HS fractions that do not penetrate the intestinal epithelium undergo phagocytosis at the site of administration. Within phagocytes, HSs are absorbed by lysosomes and hydrolytically cleaved into simpler components. The lysosomal contents are extruded into the cytoplasm, then into the circulatory system, and ultimately reach the pathological focus in the form of mono- and oligomers. This process occurs continuously as long as at least one HS molecule remains in the body.

The veterinary preparation “Tomed” (a 1% aqueous solution of peat-derived HS) in an experimental model of placental insufficiency in rats stimulated the development of adaptive processes in the afterbirth and placental bed, preventing embryo mortality and intrauterine growth restriction [205]. The activation of metabolism in the afterbirth was confirmed by an increase in the content of DNA, RNA, and glycogen in the trophoblast and amniotic epithelium. Furthermore, an increase in the relative volume of glycogen-containing cells was observed in the labyrinth and basal zone of the placenta.

In a series of experiments conducted by T.A. Zamoshchina et al. [206,207] to determine the level of adaptation to locomotor activity in a forced swimming test with a load until complete exhaustion, it was found that a five-day administration of HS prior to physical exertion increased the swimming duration of laboratory rats. The level of adaptation to physical load in animals receiving HS was comparable to the analogous indicator in rats treated with drugs of proven efficacy: Ethylmethylhydroxypyridine succinate and the complex medicinal product consisting of succinic acid, nicotinamide, riboflavin, and Riboxin [206]. Since behavioral activity is an integral indicator of functional status and reflects the organism’s response to stress, in the same experiment, animals were tested in an open field, assessing horizontal, vertical, and burrowing activity, defecation levels, and grooming. Given that HS suppress excitatory processes, a weakening of the burrowing reflex, reduced grooming episodes (evidence of a mild sedative activity of HS), and an improvement in orienting-exploratory behavior (increased rearing and horizontal motor activity) were observed in the animals [206,207].

### 2.13. Actoprotective and Nootropic Action (Preclinical Studies In Vivo)

In the work by [2], the actoprotective and nootropic effects of coal-derived HS were established. A ten-day course of intragastric administration of HS led to an improvement in the physical performance of mice in the “swimming exhaustion” test. The swimming time of animals receiving HS increased by 77.9% compared to the control group. The actoprotective activity of the HS preparation was comparable to that of “Meldonium” (*p* = 0.39), and a significant decrease in serum lactate concentration was also noted. Intraperitoneal administration of scopolamine at a dose of 1 mg/kg, 20 min prior to training sessions, resulted in pronounced central nervous system cholinergic dysfunction. A single intragastric administration of the investigated HS restored the animals’ ability to form conditioned reflexes to a level comparable to the control, induced by scopolamine-induced amnesia (*p* = 0.01). The amnesic effect of scopolamine is realized through the induction of cholinergic dysfunction in the central nervous system, suggesting that the nootropic action of HS is mediated through effects on the cholinergic system [2].

In another experiment [208], analyzing the results of biochemical blood tests, scientists concluded that HS possess nootropic and anxiolytic properties. Specifically, in biological fluid samples collected from animals after their participation in passive and active avoidance learning tests and the elevated plus maze, the following neurochemical changes were identified: enhanced central dopaminergic activity (increased concentrations of dopamine, homovanillic acid, and 3,4-dihydroxyphenylacetic acid) was observed, but serotonin turnover decreased in neurons [208].

## 3. Using Humic Matrices as Ligands for the Creation of Bionanomaterials

Due to their amphiphilic structure, which determines their self-assembly, and the large number of oxygen-containing functional groups (carboxyl, phenolic, carbonyl), HA can act as chelating agents and form complexes with various metals [209,210,211].

Given these properties, a strategy has been proposed for modulating the chemical stability and reactivity of HA, based on their conjugation with a foreign matrix consisting of organic or inorganic materials [212]. The combination of a biologically active matrix and a nanoscale core in a single entity opens up prospects for the development of a new class of multifunctional preparations with a synergistic enhancement of therapeutic properties.

Humic substances represent a promising solution as natural macromolecular ligands, they are biorefractive, polydisperse, supramolecular, and characterized by a high content of diverse functional groups. Humic substances possess natural biocompatibility and low toxicity in a wide range of concentrations, modulating cellular and systemic metabolism [213].

*Iron-containing bionanomaterials (preclinical studies in vitro and in vivo)*

A study was conducted to develop water-soluble, bioavailable iron-containing pharmaceutical substances based on nanoscale Fe(III) oxyhydroxide complexes stabilized by humic macroligands, for use as modern nanoferrotherapeutic agents for the correction of iron deficiency anemia [213]. A series of 21 samples of nanostructured complexes of HA with iron (III) were synthesized by varying the ligand type, solvent (ethanol, isopropanol, acetone), and precipitation method. Their structure, size, and morphology were characterized using ICP-AES analysis, TOC-L CSN analysis, X-ray diffraction, transmission electron microscopy, and Mössbauer spectroscopy. Additionally, their cytotoxicity (neutral red uptake method) and bioavailability (ferrozine-based colorimetric assay) were evaluated in vitro using the Caco-2 intestinal epithelial cell line. The type of humic ligand and precipitation conditions significantly influenced the physicochemical properties of the nanocomposites. The presence of humic macroligands ligands provided exceptional cytoprotection: all HS-iron(III) formulations maintained >80% cell viability and exhibited substantial bioavailability. It was established that effective membrane permeability is driven by a fine hydrophilic-lipophilic balance, which can be regulated by fractionation based on solvent action. The authors concluded that HS represent a suitable platform for iron delivery, leveraging their inherent polyfunctionality to form stable nanoclusters while demonstrating their intrinsic biocompatibility [213].

The degree of influence of iron-containing substances based on HS on hematological parameters in acute posthemorrhagic and nutritional anemia was studied [214]. The iron-containing substance samples comprised complexes of Fe(III) hydroxide with HA and FA (HA-Fe^3+^ and FA-Fe^3+^), as well as their 1:1 combinations with polymaltose (HA-PM-Fe^3+^ and FA-PM-Fe^3+^). The anti-anemic activity of these substances was investigated in 53 conventional Wistar female rats using models of acute posthemorrhagic and nutritional anemia. Anti-anemic activity was assessed by the following parameters: hemoglobin level, erythrocyte count, hematocrit, and serum iron level. The results indicated that the studied HA-Fe^3+^ and FA-Fe^3+^ samples were most effective in correcting the consequences of both experimental acute posthemorrhagic anemia and nutritional anemia. Their effect was comparable to the positive control drug, Ferrum Lek^®^ (Novartis Pharmaceutical Manufacturing, Ljubljana, Slovenia). The authors [214] concluded that the complexes of Fe(III) hydroxide stabilized by HA and FA exhibited pronounced anti-anemic activity.

*Bionanomaterials based on titanium dioxide (preclinical studies in vitro)*

Hybrid nanomaterials of HA and titanium dioxide (TiO_2_/HA-NDL) were developed, which demonstrated high antibacterial activity against Gram-negative bacteria (*Escherichia coli* DH5a, *Escherichia coli* ATCC 35218, *Klebisella pneumoniae*, *Pseudomonas aeruginosa*) and low efficacy against Gram-positive pathogens (*Enterococcus faecalis*, *Staphylococcus aureus*) [215]. Based on the results obtained using the electron paramagnetic resonance method, the researchers attributed the antibacterial properties of TiO_2_/HA-NDL to the formation of a large number of free electrons, which were stabilized and combined with TiO_2_ at a molecular level and were responsible for the generation of hydroxyl radicals and subsequent ROS-mediated damage to bacterial cells. The differences in the behavior of Gram-negative and Gram-positive strains towards the nanostructured materials were likely due to variations in the composition and structural organization of their outer bacterial membranes [215].

*Surfactant-based bionanomaterials (structural analysis)*

As a result of the interaction between HA and Pluronic F127, nanoparticles are formed that can be utilized in pharmaceuticals as carriers for hydrophobic bioactive substances, as well as antioxidants or anti-inflammatory agents [216]. The highest molar ratios of HA:PF127, specifically 1:8 and 1:80, promoted the formation of submicellar aggregates approximately 100 nm in size with zeta potentials of −28.37 and −30.23 mV, respectively. The HA–PF127 structures were spherical with a polydispersity of approximately 0.43. These results indicate that the interactions between HA and PF127 lead to the formation of stable nanoparticles [216].

*Zinc-containing bionanomaterials (preclinical studies in vivo)*

A study was conducted [217] on the wound-healing properties of zinc-containing biocompositions based on various humic ligands (HS-Zn) in an in vivo experiment using an aseptic wound model, and their resorptive properties were studied. The experiment was conducted using 70 Wistar rats on a traumatic model of a planar aseptic skin wound, and the degree of healing of the affected skin area was assessed over a period of 21 days using the planimetric method. The resorptive properties of the HS-Zn samples were studied using inductively coupled plasma mass spectrometry (ICP-MS) in biological materials (blood serum, wool, and skin from the wound surface). It was found that topical application of zinc-containing HS-Zn biocompositions over a course of treatment led to a reduction in wound area compared to the wound area treated with ZnSO_4_ at an equivalent elemental Zn concentration (1.67 mg/mL). The most pronounced regenerative effect was observed with two samples: one based on FA-Zn and another based on HA from sphagnum peat (Peat1-Zn). An increase in Zn levels was noted in the experimental skin areas of the wound surface, in the wool, and in the blood serum, indicating a resorptive action of the zinc-containing HS-Zn biocompositions during their course application, but these levels did not exceed the permissible maximum concentrations. There was also a different relationship between the tested samples, which indicates an influence of the initial HS matrix on Zn bioavailability. The authors [217] concluded that the reparative effect of zinc and humic ligand compositions, coupled with their low toxicity, warrants further investigation for the development of effective wound-healing drugs based on them.

*Silver-containing bionanomaterials (preclinical studies in vitro and in vivo)*

Most studies focus on the development of bionanomaterials based on HS and silver nanoparticles (AgNPs).

Authors V.A. Litvin and B.F. Minaev [218] synthesized nanomaterials based on AgNPs and synthetic HS as a stabilizing agent, demonstrating their antimicrobial activity against fungal (*Aspergillus niger*) and bacterial strains (both Gram-negative, *Escherichia coli*, *Pseudomonas aeruginosa*, and Gram-positive, *Staphylococcus aureus*). Under optimal reaction conditions, concentrated silver colloids (55 mM) were obtained with a yield of 99.99%, maintaining stability for over 1 year under normal conditions.

Silver nanoparticles (AgNPs) in combination with HS serve as unique depots that gradually release silver ions, creating a sustained ionic milieu that ensures prolonged drug action. The antibacterial activity of AgNPs arises from the interaction of silver ions with three primary components of bacterial cells: the peptidoglycan of the cell wall and plasma membrane, bacterial DNA, and bacterial proteins, particularly enzymes [219], and synthetic HS and HA preparations possess antimicrobial properties due to their chelating and surface-active properties, which affect the integrity of the microbial cell wall [220]. The synergistic effect exerted by silver ions and HS enhances the antibacterial properties of AgNPs, positioning them as candidates with significant potential for biomedical applications [218].

The article by Yu Zhang et al. [221] describes a one-step microwave synthesis of AgNPs utilizing natural polyelectrolytes—HS. The humic polyelectrolytes acted as chemical reducing agents for silver ions and as capping agents for the AgNPs end groups. Three sodium humates were employed: one extracted from lignite and leonardite, and one sodium fulvate isolated from natural brown water that had percolated through peat deposits. It was established that both conventional and microwave synthesis using coal humates resulted in the formation of small-sized AgNPs ranging from 4 to 14 nm, with the majority of particles measuring (6 ± 2) nm as assessed by TEM. Peat fulvate yielded much larger AgNPs, ranging from 10 to 50 nm according to TEM evaluation. A significant acceleration of reaction time—by 60–70 times—was achieved using microwave irradiation: from 240 min down to 210–240 s. Coal humate-stabilized AgNPs exhibited antimicrobial properties against *S. aureus*. The authors [221] concluded that microwave synthesis offers substantial advantages in terms of time and scalability for the large-scale production of AgNP-HS preparations with antimicrobial properties, suitable for topical application in wound healing.

The article by M.V. Zykova et al. [125] describes a synergistic synthetic approach for obtaining new and effective biomedical products that combine the “green” properties of HS with nanoscale silver forms. The study’s objective was to evaluate the AO activity of HS matrices (ligands forming nanoparticles) and active substances (biomaterials) based on ultrafine HS-stabilized AgNPs (HS-AgNP). In vitro cytotoxicity studies (using the 3T3-L1 fibroblast cell line) indicated that the conjugation of AgNPs with the parent HS matrices prevents excessive cytotoxicity of nanoscale silver compositions. Through in vitro experiments (the ABTS assay and the 3T3-L1 fibroblast cell line assay using the fluorescent probe DCFDA, both without added prooxidant and with hydrogen peroxide (H_2_O_2_)-stimulated intracellular ROS production), it was discovered that all parent HS matrix samples, as well as HS-AgNP, exhibited no pro-oxidant effects and possessed high AO activity. Notably, all HS-AgNP samples displayed greater AO activity than their parent HS matrices. Two proposed mechanisms for the pronounced AO activity of HS-AgNP were suggested: firstly, the inherent robust capacity of HS to inactivate ROS, and secondly, the larger surface area and higher surface-to-volume ratio of HS-AgNP, which facilitate electron transfer and reduce kinetic barriers for the reduction reaction. The authors [125] concluded that the new HS-AgNP biomaterials are of particular interest for research aimed at inhibiting bacterial and viral growth and promoting the healing of suppurating wounds. Similarly, high AO activity of HS-AgNP biomaterials was observed in experiments using tert-butyl hydroperoxide as an oxidant to stimulate ROS production in a culture of normal 3T3-L1 fibroblast cells in vitro [222].

An in vitro study using peritoneal macrophage cell cultures was conducted to investigate the cytotoxic, pyrogenic, and immunomodulatory properties (via the arginine balance) of bio-nanomaterials based on AgNPs ultradispersed in matrices of HS (HS-AgNP), as well as their effect on the pro- and anti-inflammatory properties of antigen-presenting cells [223]. It was demonstrated that native humic matrices promote the polarization of peritoneal macrophages towards the classical M1 phenotype by enhancing NO-synthase activity and inhibiting arginase. In contrast, the HS-AgNP bio-nanomaterials modulated an alternative M2 or an “M2-like” activation state of macrophages (M2-like state). The HS were non-cytotoxic at effective concentrations, and three out of the four tested samples were free of pyrogenic impurities. The authors [223] concluded that the use of HS and silver-containing HS-based bio-nanocomposites, which possess a broad ability to influence the polarization of antigen-presenting cells, represents a promising research direction for modulating inflammatory responses and, specifically, for addressing the acute socio-medical problem of chronic wound treatment. A similar study was performed for humic matrices modified with 2-hydroquinone and 2-hydroxynaphthoquinone and the resulting HS-AgNP bio-nanomaterials [224]. It was established that the modified HS-AgNP samples specifically enhance the M2 properties of peritoneal macrophages by inhibiting NO-synthase and significantly activating arginase, thereby reinforcing the anti-inflammatory properties of the cells. These samples lacked cytotoxic effects and were free of pyrogenic impurities. The investigated HS-AgNP samples were shown to influence immune response mechanisms and represent an effective approach for correcting inflammation and promoting regeneration [224].

To address the problem of antibiotic resistance, the use of bio-nanomaterials based on silver nanoparticles ultradispersed in a coal-derived HS matrix is proposed [225], synthesized through various modifications of the HS structure using hydroquinone, 2-methylhydroquinone, pyrocatechol, 1,4-naphthoquinone, and 2-hydroxy-1,4-naphthoquinone (using Fenton reaction and phenol-formaldehyde copolycondensation), resulting in a total of 12 derivatives. It utilized several opportunistic microorganisms: standard strains (*Escherichia coli* ATCC 25922; *Staphylococcus aureus* ATCC 25923; *Methicillin-resistant Staphylococcus aureus* (MRSA) ATCC 33592; *Klebsiella pneumonia* ATCC 700603; *Pseudomonas aeruginosa* ATCC 9027) and clinical isolates of these bacterial species, obtained from various patient specimens (urine, sputum, wound, uterine cavity, blood). A series of experiments were conducted: assessment of biofilm-forming activity, determination of microbial susceptibility to these bio-nanomaterials, analysis of changes in crystal violet uptake by the cell wall, and evaluation of bacterial viability. The results established that all bio-nanomaterials based on ultradispersed silver nanoparticles within the HS matrix exhibited antibacterial activity against all tested opportunistic pathogens. They also demonstrated an inhibitory effect on both the process of biofilm formation and pre-formed biofilms. The sample CHP-pHQ-FE-Ag (derived from the *p-*hydroquinone-modified HS matrix synthesized via the Fenton reaction) exhibited the highest antibacterial activity against Gram-negative strains (*E. coli*, *A. baumannii*, *K. pneumoniae*, *P. aeruginosa*). The highest antibacterial activity against Gram-positive strains (*S. aureus*, *MRSA*) was demonstrated by the sample CHP-AgNPs-MW (the base matrix is unmodified coal-derived HS, where the HA-AgNPs sample was obtained via microwave-assisted synthesis) [225].

A study was conducted to investigate the restoration of lincomycin sensitivity in a *Methicillin-resistant Staphylococcus aureus* strain following the addition of bio-nanomaterials based on AgNPs. These nanoparticles were ultradispersed within a matrix of coal-derived HS and a hydroquinone-modified HS sample [226]. The sensitivity of standard and clinical strains of *Methicillin-resistant S. aureus* was determined by measuring the MIC for compositions containing lincomycin and tetracycline. High sensitivity to tetracycline (MIC < 10 µg/mL) and no sensitivity to lincomycin (MIC > 200 µg/mL) were established. After the addition of the tested AgNPs samples to lincomycin, high sensitivity of *S. aureus* was observed. Specifically, the MIC for the composition of lincomycin + hydroquinone-modified HS and AgNPs (40 µg/mL) and the composition of lincomycin + HS and AgNPs (60 µg/mL) was less than 0.1 µg/mL. Thus, a complete restoration of lincomycin sensitivity in the *Methicillin-resistant S. aureus* strain was achieved. The authors [226] concluded that the mechanism of the synergistic action of HS-stabilized nanosilver and lincomycin can be attributed to the formation of labile surface complexes between HS and lincomycin. These complexes may dissociate and release the antibiotic upon the intracellular uptake of the silver nanoparticles. Thereby, a new way for combating antibiotic resistance is unveiled.

## 4. Safety of Using Humic Substances (Preclinical Studies In Vitro and In Vivo)

Numerous scientific studies have demonstrated that HSs are safe, non-teratogenic, non-mutagenic, do not induce allergic reactions, and exhibit no sensitizing or irritant properties [2,51,184,227,228,229,230,231]. They are characterized by very low toxicity and high LD_50_ values [51,232].

In the study by C.E.J. van Rensburg et al. [51], Sprague-Dawley rats were subjected to daily oral treatment with a humate derived from leonardite at a dose of 1000 mg/kg body weight for 1 month. Blood samples were collected from the animals at the beginning and end of the study and the organs (liver, kidneys, spleen) were examined for any anomalies after the treatment. Furthermore, a teratogenicity study was conducted in which pregnant rats were administered the test substance at a dose of 500 mg/kg body weight from day 5 to day 17 of pregnancy. The pups were monitored for clinical and behavioral abnormalities for 2 weeks after birth. All animals were weighed daily, and observed for pain and distress. No signs of toxicity were observed, no animals died, and no significant changes were noted in the hematological profiles of the experimental group before and after treatment, or any abnormalities in the rat pups.

The study [21] also reports the harmlessness and non-toxicity of HS based on findings from investigations into acute and chronic toxicity, accumulation, mutagenicity, embryotropic activity, teratogenic effects, embryotoxicity, and irritant and resorptive effects of HS preparations. Toxicity studies indicated that potassium humate is safe for human consumption at a daily dosage of up to 1 g/kg, and FA is safe at a daily dosage of up to 1.8 g/kg [19].

Works focusing on the TPP have not identified any embryotoxic or teratogenic effects (in experiments with hamsters and rats, at a dose range of 5 to 50 mg/kg [230], nor mutagenic or genotoxic properties [229], HS did not induce or enhance allergic sensitization (in experiments with mice and guinea pigs) [227,228].

When HA were administered to rats at a deliberately lethal dose (480 mg/kg, raperitoneal route of administration), it was observed [232] that animal mortality resulted from acute heart failure, stemming from ischemic myocardial dystrophy. A decrease in cardiac electrical stability was noted, manifesting as a reduction in the ventricular fibrillation threshold (1.8-fold) and a shortening (2.8-fold) of the QRS complex compared to intact animals. This suggests that at therapeutic doses, HA can exert cardiotropic effects, a finding corroborated by the authors of this experiment in several scientific studies [152,153].

An assessment of the cytotoxic effects of coal-derived HS on the HepG2 cell line, using the MTT assay after 24 h of incubation, showed inhibition of cell culture viability only at the highest concentrations, the IC_50_ value for the HS preparation was 5251 ± 621.7 µg/mL, indicating an absence of significant cytotoxic activity [2]. The results of acute and chronic toxicity evaluations, as well as the allergenic potential of the same coal-derived HS preparation, indicated that a single intragastric administration of the investigated HS sample at a dose of 2000 mg/kg did not lead to animal mortality, either on the day of administration or during the subsequent 14-day observation period, and there was no effect on the body weight dynamics of the animals. No clinical signs of health impairment were recorded, and macroscopic signs of internal organ damage were not found upon dissection of animals on day 15, clinical signs of impairment were also not observed after a 28-day course of intragastric administration of HS at a dose of 1000 mg/kg. There were no changes in animal body weight dynamics or food consumption, serum hematological and biochemical parameters did not differ from control values, and kidney metabolic parameters remained unchanged. Macroscopic and histological examinations revealed no pathological changes in organs and tissues. During the assessment of the sensitizing effects of the investigated HS sample when applied to the skin of guinea pigs after 10 and 20 applications, no cutaneous reaction was observed at the application site [2].

The absence of irritant and sensitizing effects of HA (at various concentrations up to 10%) was also demonstrated in the study by K. Wiegleb et al. [233] using the HET-CAM test. The work by C.E.J. Van Rensburg et al. [78] also note the absence of toxic effects from a 5.3% oxifulvic acid-based cream in experimental animals following acute and/or subchronic application.

The 90-day toxicity of HA was investigated in the work by T.S. Murbach et al. [234]. Wistar rats were administered an HA preparation derived from lignite intragastrically at doses of 0.5; 1000 and 2000 mg/kg body weight daily for 90 days. Observations were made regarding mortality, clinical signs, behavior, body weight, and ophthalmological changes. A series of functional observations were conducted during the final week of treatment. Post-treatment, blood samples were collected from the animals, macroscopic pathological examination of organs and their weights were performed, along with histopathological examination of organ and tissue samples. No general or organ-specific toxicity and no adverse effects were observed in rats after the 90-day course. The authors also found that the tested preparation exhibited no genotoxic potential in the bacterial reverse mutation test, did not induce chromosomal aberrations in mammalian cells in vitro, and did not induce micronucleus formation in mouse bone marrow erythrocytes in vivo after repeated intragastric administration at doses of 0.5; 1000 and 2000 mg/kg body weight.

Toxicity studies of FA conducted by J.J. Gandy et al. indicated its safety at doses up to 400 mg/kg body weight, corresponding to hazard class 4 [235], studies on acute and chronic toxicity, as well as wound healing effects, proved the safety of this FA sample [54,235].

A large number of scientific studies have focused on investigating the antimutagenic and desmutagenic activity of HS. In the article by G. Ferrara et al. [236], the antimutagenic/desmutagenic activity of HA isolated from leonardite and soil was studied on the human lymphoblastoid cell line TK6, treated with mitomycin C as a reference mutagen, using the micronucleus induction assay. Neither of the HA used individually exhibited genotoxic effects. However, both HA significantly reduced (*p* ≤ 0.001) the number of micronuclei induced by mitomycin C. Furthermore, the studied HA showed a mild protective effect against the cytotoxicity of mitomycin C towards TK6 cells. Humic acids isolated from leonardite demonstrated a more pronounced desmutagenic/antimutagenic activity, possibly due to a higher content of carboxyl groups and a lower content of phenolic groups [236]. A similar study [237] was performed on Chinese hamster ovary cells in vitro, both individually and in combination with two mutagens (mitomycin C and maleic hydrazide). Data on sister chromatid exchange, mitotic indices, and proliferation indices were collected, and the authors noted the desmutagenic activity of HS.

I. Marova et al. [238] investigated the antimutagenic effects of HA derivatives from various sources using the yeast strain *Saccharomyces cerevisiae D7*, the study assessed their ability to inhibit the formation of mutant colonies in the presence of the standard mutagen 4-nitroquinoline-N-oxide. All tested humates demonstrated antimutagenic activity, with the exception of sodium humate obtained at 250 °C. This particular humate exhibited a genotoxic effect, which the authors attributed to the accelerated formation of ROS within the HA molecule at the higher temperature [238].

Previously, the effects of HS to prevent the damaging effects of heavy and toxic metals were also noted [183,184,185,186,187,188,189,190,194,195]. This is due to the fact that HS containing various functional groups in their structure, with a large variability in their mutual arrangement relative to each other, represent unique high-molecular ligands capable of coordinating various acceptor ions and molecules on their surface. Nitrogen-containing functional groups of HS are softer ligands and are suitable for the strong binding of softer ions such as Ag^+^, Fe^2+^, Cd^2+^, Cu^2+^, and others. Oxygen-containing residues of carboxyl and phenolic groups, as harder ligands, more strongly bind harder ions such as Fe^3+^, Al^3+^, Mg^2+^, and others. The steric accessibility of the atomic orbitals of the donor atoms of adjacent functional groups of HS enables the formation of mixed-ligand and polynuclear complexes. Moreover, the large number of unsaturated aromatic bonds in the humic structure enables the formation of π-complexes with transition metal ions and other low-molecular-weight unsaturated compounds. The diversity of ligand fragments in the humic structure of HS makes them unique, enabling the formation of both highly stable and labile complexes with various metals, which ensures either strong metal binding or, conversely, rapid metal exchange. This is also a key safety factor for the use of bionanomaterials based on HS and metal nanoparticles. It is the presence of humic ligands that ensures the safety of such medicinal molecules [125,213,217,222,223,224].

## 5. Conclusions

The multifunctionality of HS is their fundamental property. The presence of a large number of different functional groups in their molecules ensures their ability to participate in ligand exchange and heterogeneous processes, forming a variety of intra- and intermolecular bonds that determine their redox, chelating, and protolytic properties. Humic substances are characterized as some of the most powerful chelating agents among natural organic substances; their zwitterionic nature allows for various interactions involving anions and cations. The unique chemical properties of HSs enable them to function as buffer systems capable of regulating protolytic balance in various biological environments and also act as free radical scavengers. All these properties are key to the manifestation of biological activity and determine the unique chemical and pharmacological potential of HS.

Numerous studies have demonstrated the positive effects of HS on various aspects of biological system functioning and lack of their toxicity. The unique chemical structure of HS defines their multifaceted pharmacological activity and it does not indicate non-specificity in their biological action. The pleiotropic effects of HS are the result of cascade reactions in the body caused by their influence on physiological systems activated by the redox, acid-base, and macrocolloidal properties of HS and their nonspecific ligand-receptor interactions. Particularly important among them is the influence of humic substances on the body’s antioxidant defense system and the immune system. Due to this, HSs have been described as exhibiting the following pharmacological effects: immunotropic, anti-inflammatory, anti-allergic, antibacterial, antifungal, antiviral and antitumor actions, antioxidant and antiradical activity, cardioprotective, hepatoprotective, nephroprotective, neuroprotective, gastroprotective, cytoprotective, antihypoxic, antidiabetic, regenerative, antitoxic, adaptogenic, actoprotective and nootropic actions, biostimulating effects, wound-healing properties, a positive effect on the microbiome and the bioavailability of medicinal molecules. Humic substances are unique and effective macroligands for the creation of new-generation bionanomaterials with high pharmacological selectivity and no toxic effects.

Given such facts as the multifaceted pharmacological activity and the lack of strict consistency of chemical composition, the impossibility of predicting their biological activity without experimentation remains a challenging aspect of HS research today. This review presents a large body of literature data, illustrating that HSs, in each specific case, can be sources of new and diverse biologically active molecules depending on the type and origin of the raw material source, the method of HS isolation, the extractants used, the temperature regime, the methods of structural modification, etc. This fact necessitates the ongoing biological and chemical standardization of each specific HS sample. Therefore, it is impossible to unify each HS sample with respect to any type of biological activity, meaning that absolutely every new HS molecule must be subjected to pharmacological testing.

It is also very important to note, in nature, compounds with such a combination of important chemical and biological properties are not found. Therefore, HSs are promising biologically active substances for the discovery and development of next-generation pharmaceuticals. Alongside the creation of highly effective synthetic drugs, there is a need to search for new natural compounds that are not inferior in efficacy to chemical drugs but possess low toxicity and lack side effects. This is due to the fact that the increase in the concentration of xenobiotics in the environment, food products, and water, associated with societal industrialization, causes significant damage to human health. Upon entering the body, xenobiotics either directly exert negative effects or, through biotransformation, form toxic metabolites that lead to undesirable consequences. Most pharmaceutical drugs are also products of chemical synthesis, which, upon administration, increase the toxic load on the body and cause a wide range of side effects. In this context, HSs are precisely such promising natural substances with a wide range of biological activity, high efficacy and specificity of action, and absence of toxicity. Undoubtedly, pharmaceuticals based on HS can offer strong competition to synthetic drugs that possess a considerable set of side effects.

## Data Availability

No new data were created or analyzed in this study. Data sharing is not applicable.
